# *De novo* transcriptome assembly and discovery of drought-responsive genes in white spruce (*Picea glauca*)

**DOI:** 10.1371/journal.pone.0316661

**Published:** 2025-01-03

**Authors:** Zoé Ribeyre, Claire Depardieu, Julien Prunier, Gervais Pelletier, Geneviève J. Parent, John Mackay, Arnaud Droit, Jean Bousquet, Philippe Nolet, Christian Messier

**Affiliations:** 1 Département des Sciences Naturelles, Institut des Sciences de la Forêt Tempérée (ISFORT), Université du Québec en Outaouais (UQO), Ripon, Canada; 2 Centre d’étude de la Forêt (CEF), Québec, QC, Canada; 3 Canada Research Chair in Forest Genomics, Institute for Systems and Integrative Biology, Université Laval, Québec, QC, Canada; 4 Centre for Forest Research, Département des Sciences du Bois et de la Forêt, Université Laval, Québec, QC, Canada; 5 Natural Resources Canada, Canadian Forest Service, Laurentian Forestry Center, Québec, QC, Canada; 6 Plateforme de Bioinformatique du Centre Hospitalier Universitaire de Québec Associé à l’Université Laval, Québec, QC, Canada; 7 Laboratory of Genomics, Maurice- Lamontagne Institute, Fisheries and Oceans Canada, Mont-Joli, QC, Canada; 8 Department of Plant Sciences, University of Oxford, Oxford, United Kingdom; 9 Département des Sciences Biologiques, Université du Québec à Montréal (UQAM), Montréal, QC, Canada; ICIFOR-INIA, CSIC, SPAIN

## Abstract

Forests face an escalating threat from the increasing frequency of extreme drought events driven by climate change. To address this challenge, it is crucial to understand how widely distributed species of economic or ecological importance may respond to drought stress. In this study, we examined the transcriptome of white spruce (*Picea glauca* (Moench) Voss) to identify key genes and metabolic pathways involved in the species’ response to water stress. We assembled a *de novo* transcriptome, performed differential gene expression analyses at four time points over 22 days during a controlled drought stress experiment involving 2-year-old plants and three genetically distinct clones, and conducted gene enrichment analyses. The transcriptome assembly and gene expression analysis identified a total of 33,287 transcripts corresponding to 18,934 annotated unique genes, including 4,425 genes that are uniquely responsive to drought. Many transcripts that had predicted functions associated with photosynthesis, cell wall organization, and water transport were down-regulated under drought conditions, while transcripts linked to abscisic acid response and defense response were up-regulated. Our study highlights a previously uncharacterized effect of drought stress on lipid metabolism genes in conifers and significant changes in the expression of several transcription factors, suggesting a regulatory response potentially linked to drought response or acclimation. Our research represents a fundamental step in unraveling the molecular mechanisms underlying short-term drought responses in white spruce seedlings. In addition, it provides a valuable source of new genetic data that could contribute to genetic selection strategies aimed at enhancing the drought resistance and resilience of white spruce to changing climates.

## 1. Introduction

Climate change projections raise concerns about trees having to cope with intensified and frequent extreme events [1]. Drought is currently causing heightened disruptions in forests, diminishing resilience and increasing mortality rates [2]. Future climates may reduce the productivity of essential conifer species in forests, underscoring the importance of prioritizing resilient and productive species for warmer, drier conditions. In this regard, recent research efforts started to look at methods and approaches for the selection and breeding of more resilient conifers (e.g., Depardieu et al., 2020 [3]; Laverdière et al., 2022 [4]; Soro et al., 2023 [5]). However, in spite of recent progress [6–9], there are still large gaps in our understanding of the complex molecular response of trees to drought at the transcriptome-wide level.

Drought response in long-lived woody plants such as conifers involves an complex network of genes and molecular mechanisms [10]. Indeed, several major gene classes or families are involved in short-term physiological responses to drought, including cell growth, photosynthesis, water loss, phytohormones metabolism, stomatal aperture and closure, and the maintenance of osmotic balance [11]. More specifically, the pivotal role of the phytohormone ABA as a precursor molecule in drought stress signaling is widely acknowledged in coniferous species [8]. In addition, aquaporins (AQPs) and ion channels facilitate the transport of water and ions across cell membranes, playing a critical role in regulating water balance in trees [12]. Previous studies have revealed modifications in the regulation of genes responsible for synthesizing and transporting defense molecules such as flavonoids and terpenoids [7, 13], antioxidants involved in ROS scavenging [14, 15], osmoprotectants such as carbohydrates [16, 17], and proline [18, 19]. Heat shock proteins (HSPs) play a pivotal role in safeguarding and stabilizing proteins under drought conditions [17]. Similarly, chaperone proteins like dehydrins, a subset of late embryogenesis abundant (LEA) proteins, maintain protein and cell membrane stability throughout the hydric constraint [6, 20]. In such conditions, alterations in the composition and structure of the cell wall are directed by the control of genes linked to the synthesis of cell wall polysaccharides and membrane [21, 22]. Finally, genes involved in transcriptional regulation networks, such as AP2/ERF, bZIP, TCP, WRKY, and MYB transcription factors, coordinate molecular responses to drought [15, 23–25].

White spruce (*Picea glauca* (Moench) Voss) is a conifer species widely distributed across Canada and the northern USA, known for its straight grained and strong wood, making it valuable for lumber and pulp production [26, 27] in addition to its ecological importance. Its rapid growth in various environments makes white spruce an important species in forestry and reforestation efforts, representing a significant portion of Canada’s forest inventory and being one of the most widely planted tree species [26, 28]. It is also considered to be a model conifer species for genetic and genomic investigations [29]; several studies have shown the susceptibility of white spruce to drought, as demonstrated by a marked reduction in growth [3, 5, 30, 31], increased mortality [32, 33], and changes in population abundance and distribution [34]. Similarly to numerous conifers of the *Pinophyta* group, white spruce swiftly initiates the ABA pathway under drought conditions, leading to early stomatal closure [35]. This process reduces water loss while also concurrently decreasing photosynthetic uptake, thereby posing a risk of carbohydrate depletion if the drought persists [36]. The importance of intraspecific genetic variation for drought response has also been recently highlighted in white spruce [3, 37]. Thus, characterization of its intraspecific variability at the molecular level appears essential to better delineate the tolerance threshold of stress and identify potential genetic traits governing a tree species’ drought response and resilience [38]. Understanding white spruce’s molecular response to drought is crucial for elucidating acclimation and adaptation mechanisms, informing sustainable forest management, and enhancing resilience to changing climates [29].

Recent studies have identified genes associated with drought adaptation in white spruce based on testing extensive lists of candidate genes rather than the entire transcriptome [7, 39]. Despite the rapid proliferation of genomic resources, including nuclear [40, 41], mitochondrial, and chloroplast genomes [42], gene catalogs [43], SNP catalogs [44–46], and quantitative trait loci (QTL) analyses [13, 47, 48] brought about by advancements in Next Generation Sequencing (NGS) and high-throughput genotyping technologies, these resources still remain incomplete and fragmented [49, 50]. Considering the extensive gene flow linking natural populations of white spruce [51], the relatively recent nature of local genetic adaptation to climate following Holocene recolonization [52], and the highly multigenic nature of local adaptation to climate in spruces [39, 52], it is anticipated that there may be dozens to hundreds of genes potentially involved in drought response and resilience. Consequently, it is imperative that genome-wide and/or transcriptome-wide studies are conducted to elucidate the molecular bases of these polygenic traits more comprehensively.

This study was carried out in white spruce and had three main objectives. First, we assembled a *de novo* transcriptome based on RNA sequencing (RNA-Seq) of samples sourced from distinct developmental stages of white spruce and subjected to short and long-term drought and to defoliation. This approach aimed to capture a broad sampling of expressed genes, specifically emphasizing the response to drought conditions. Second, transcriptomic analyses were conducted on the foliage of white spruce seedlings during a 22-day greenhouse drought experiment to identify key drought-responsive genes involved in short-term water stress acclimation in this species. Third, enrichment analyses of differentially expressed genes were performed to highlight the main metabolic pathways involved in response to short-term water stress. We also exploring the intraspecific variation in drought-responsive genes using three clones. Given the large hydraulic safety margin of white spruce and its drastic reduction in gas exchange during drought [31], we hypothesize that under severe and short-term drought conditions, this species will prioritize the regulation of water management processes from the onset of treatment, at the expense of growth-related processes. Consequently, we expect to observe an up-regulation of genes involved in water homeostasis and transport, alongside a down-regulation of genes associated with photosynthesis and growth.

## 2. Material and methods

### 2.1. *De novo* transcriptome assembly and functional annotation of the white spruce transcriptome assembly, GCAT 4.0

#### 2.1.1. Plant material

A *de novo* transcriptome was assembled from RNA-Seq data obtained from three distinct experiments involving the collection of *Picea glauca* foliage. A total of 16 samples came from a common garden experiment belonging to the International Diversity Experiment Network with Trees (IDENT) network, where eight trees had been subjected to water exclusion and eight others to summer irrigation since 2014 ("Experiment 1", see S1 Methods and S1 Table in Supporting information for details). The access of IDENT site and sampling permission was provided by the Forest Research and Monitoring Section of the Ontario Forest Research Institute. Six other samples came from a greenhouse experiment with a budworm-induced biotic stress treatment ("Experiment 2"; S1 Methods and S1 Table in Supporting information). The inclusion of data from Experiment 2 was motivated by the reported points of convergence in the signalling networks involved in responses to abiotic and biotic stresses in plants [53, 54]. Six samples were from a greenhouse drought stress experiment on young clonal seedlings including three water-stressed and three well-watered seedlings (control seedlings in "Experiment 3"; S1 Methods and S1 Table in Supporting information), as previously described in Stival Sena et al. (2018) [6]. A total of 28 samples were used for *de novo* transcriptome assembly. Information about sample pool collections, RNA extraction and integrity assessment, library construction, and sequencing can be found in the Supporting Information file (S1 Methods and S1 Table).

#### 2.1.2. RNA *de novo* transcriptome assembly

Quality of RNA-seq raw sequence data was first checked using FASTQC v0.11.9 [55]. Raw reads were cleaned using Trimmomatics.0.39 [56] to remove poorly sequenced nucleotides and remaining adaptor sequences. Clean reads were further filtered for length longer than 30 bp. For each of the 28 samples, filtered reads were used to produce a transcriptome assembly using the A5 pipeline [57] that integrate an overlap-based assembler to correct base-call errors (SGA tool [58]) and a de Bruijn graph assembler (IDBA-UD [59]) to produce contigs that are latter scaffolded using SSPACE [60]. Transcriptome assemblies were then scaffolded with one another using LINKS 1.8.6 [61]. The resulting consensus assembly was then scaffolded again with a previously published *Picea glauca* transcriptome assembly [43] using LINKS 1.8.6 and sequences shorter than 500 bp were removed as they were not likely to code for functional proteins. The completeness of this new assembly, hereafter named GCAT 4.0, was then evaluated using BUSCO (Benchmarking Universal Single-Copy Orthologs) v5.4.3 with -m transcriptome option and sequence comparison with the *Embryophyta* and *Viridiplantae* reference databases (odb10) [62]. The number of open reading frames (ORFs) and other complementary statistics were performed using the TRAPID web server (https://bioinformatics.psb.ugent.be/trapid_02/) and the PLAZA version 4.5 database (https://bioinformatics.psb.ugent.be/plaza/versions/plaza_v4_5_dicots/) [63].

#### 2.1.3. Functional annotation of transcripts

Functional annotation for the new transcriptome assembly was retrieved by sequence similarity searches using BLASTx of OmicsBox [64, 65] (cut-off E-value of ≤10^−5^) against the Refseq database from the NCBI (Accessed September 29th, 2022). The description of protein signatures was obtained after detection of homologous protein domains of translated sequences following a search of the Interpro database using the OmicsBox. Gene Ontology (GO) annotations including GO molecular function, GO biological process and GO cellular component terms were also obtained for each individual transcript using GO Annotation tool in OmicsBox. To obtain a complete annotation of the *de novo* assembly GCAT 4.0, BLASTx analyses were performed against public databases such as PlantTFDB and *Viridiplantae*, using DIAMOND-aligner v.2.0.14 [66]. Analysis parameters were set to "sensitive" mode, k-1, b1.2 and an E-value of ≤10^−5^. The OmicsBox assembly annotation has been deposited and is publicly available (https://github.com/ZoeRibeyre/De-novo-transcriptome-assembly-and-discovery-of-drought-responsive-genes-in-white-spruce.git). Putative genes were annotated against the transcriptomes of *Arabidopsis thaliana*, *Populus trichocarpa and Malus domestica* (data downloaded from PLAZA 5.0, sub-sections Locus FASTA Data—Protein files—Selected transcript [67]) using Blastx with an E-value cut-off set to ≤10^−5^.

The presence of transcription factors (TFs) was additionally corroborated by analyzing BLASTx results against the plant transcription factor database PlantRegMap/PlantTFDB v5.0 (http://planttfdb.gao-lab.org/; [68, 69] and the Refseq database, and based on protein signatures detected using OmicsBox. The number of putative unique genes contained in the *de novo* transcriptome assembly GCAT 4.0 was determined by BLASTn analysis against the latest white spruce reference genome publicly available on NCBI (WS77111v2, Accessed on July 2022).

### 2.2. Drought stress experiment and transcriptome analysis

#### 2.2.1. Plant material, water treatment, and RNA sequencing

**Fig 1:**
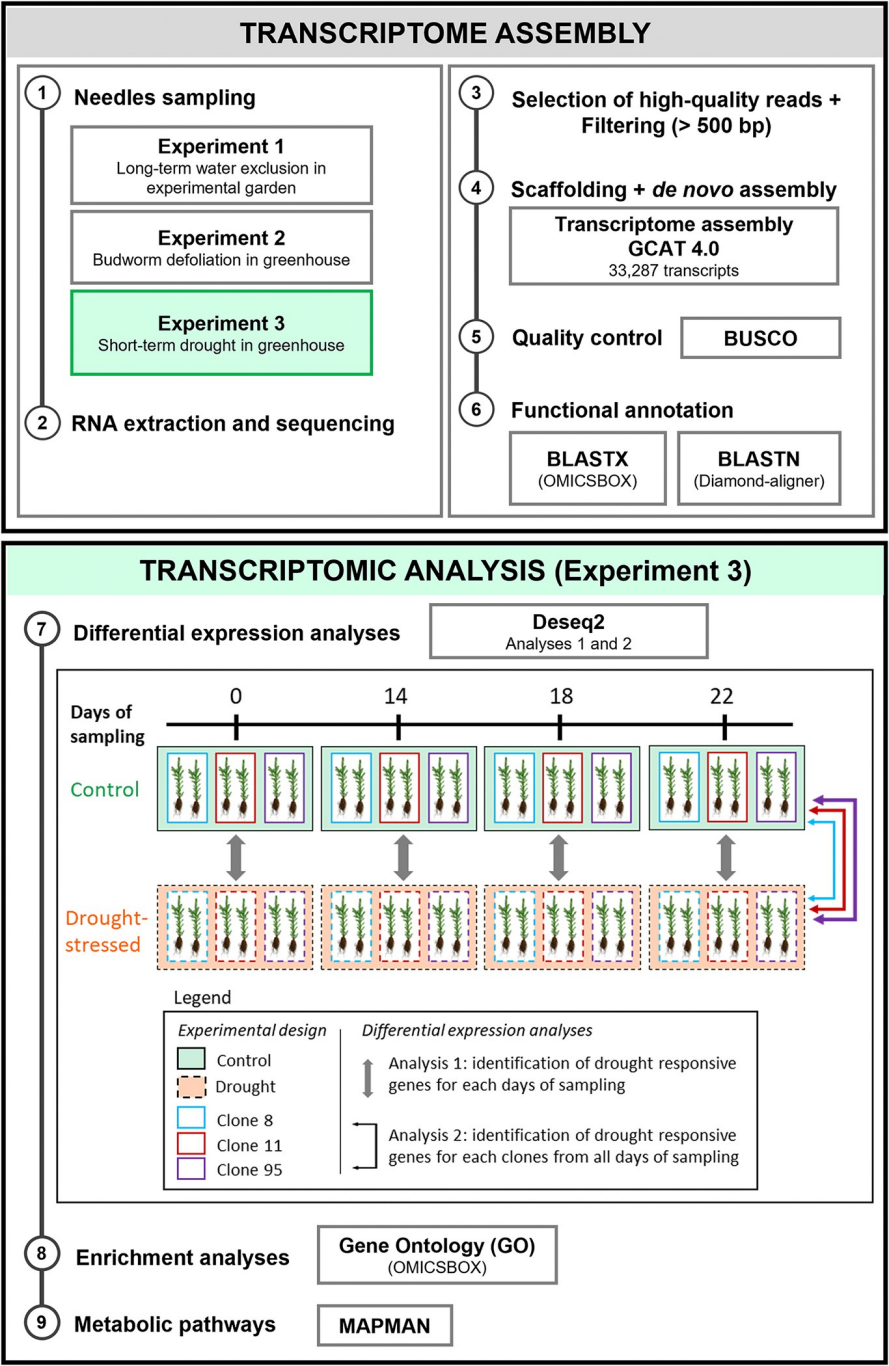
Experimental design and analysis pipeline used in this study. The analysis steps are presented in chronological order for the two boxes Transcriptome assembly and Transcriptomic analyses. The tool used for each type of analysis is reported.

Transcriptomic analyses, composed of differential expression and enrichment analyses, were performed on raw RNA-seq data from 2-year-old white spruce foliage submitted to a greenhouse water stress experiment published by Stival Sena et al. (2018) [6] (referred to in this study as "Experiment 3"). The seedlings were represented by three genetically unrelated 2-year-old clones (C8, C11, and C95). The seedlings were watered twice per week for two months before the experiment. Then, following a completely randomized design, half of the plants were watered (controls) and the other half were withheld from water (stressed) for 22 days. The newly formed foliage (needles) was sampled at 0, 14, 18, and 22 days (6 samples per condition and time point, n = 48) (see Experiment 3, Fig 1 and S1 Table). Needles were frozen in liquid nitrogen immediately after sampling and stored at −80°C until RNA extraction. At each sampling day, the midday water potential (branch) of four plants per genotype in control and stressed treatments was measured using a Scholander pressure chamber (Model 610, PMS Instruments, Albany, OR, USA). Water potential measurements show a sharp decline starting on day 14, which intensifies on day 18 and even further by day 22, reflecting both the severity of the imposed drought and the trees’ physiological response to this stress (see Sena Stival et al., 2018). More detailed information about sample pool collections, RNA extraction and integrity assessment, library construction, and sequencing can be found in the Supporting Information file, specifically in S1 Methods and S1 Table, within the Experiment 3 section.

#### 2.2.2. Differential expression and enrichment analyses

Differential expression analyses between drought-stressed and control seedlings were carried out to identify transcripts involved in conifer drought response. A first set of analyses was carried out at each time point (Analysis 1, Fig 1) to identify the transcripts significantly up- or down-regulated throughout drought intensification. Considering the insufficient number of replicates to perform a clone-by-clone analysis for each time point, the transcripts differentially expressed for each clone were determined by comparing water stress versus control conditions for all time points (Analysis 2, Fig 1). Differential expression analyses were conducted by pseudo-aligning high-quality reads against the assembly GCAT 4.0 using Kallisto v0.48.0 [70]. Read counts were normalized using DESeq2 [71] and DESeq-normalized expression values were then used to calculate the fold change for a given transcript expressed as a log2-fold change (LFC). Differentially expressed transcripts (DETs), and corresponding genes (DEGs), between drought-treated and control samples were then identified using the R package DESeq2 [71] with an absolute threshold of 2 for LFC and an adjusted p-value of 0.05.

Gene ontology (GO) enrichment analyses were performed on significant DETs using the OmicsBox and a Fisher’s exact test [64]. GO enrichment analyses were based on lists of DETs whose expression was significantly regulated at each time point. Venn diagrams were generated to highlight unique and shared DEGs between time points and clones using Venn diagrams (ggVennDiagram v1.2.2 R package, [72]; Venndetail v1.16.0 R package, [73].

The functions and the regulation of DEGs were visualized using metabolic pathway diagrams from MapMan v3.6.0RC1 [74, 75]. MapMan manages a hierarchical tree structure that describes different functional categories or "Bins" according to the MapMan nomenclature. The Mercator4 online tool was used to create the mapping file required to run MapMan from a FASTA file (the *de novo* assembly transcriptome GCAT 4.0) by assigning sequences to the corresponding Bin terms [76]. This analysis was conducted at the gene level. All available metabolic pathway diagrams were downloaded from the MapMan interface and visualized following the analyses. We selected the pathway diagrams for metabolism (X4.5 Metabolism Overview R5.0) and photosynthesis (X4.5 Photosynthesis R5.0) as those had the most DEGs identified in our study. To improve understanding of the results in the context of drought-related response, we graphically synthesized parts from the Cellular_response_overview pathway and abiotic results from the Biotic Stress pathway by removing sections with very few or no DEGs.

## 3. Results

### 3.1. Statistics and quality assessment of the new white spruce *de novo* assembly, GCAT 4.0

**Fig 2:**
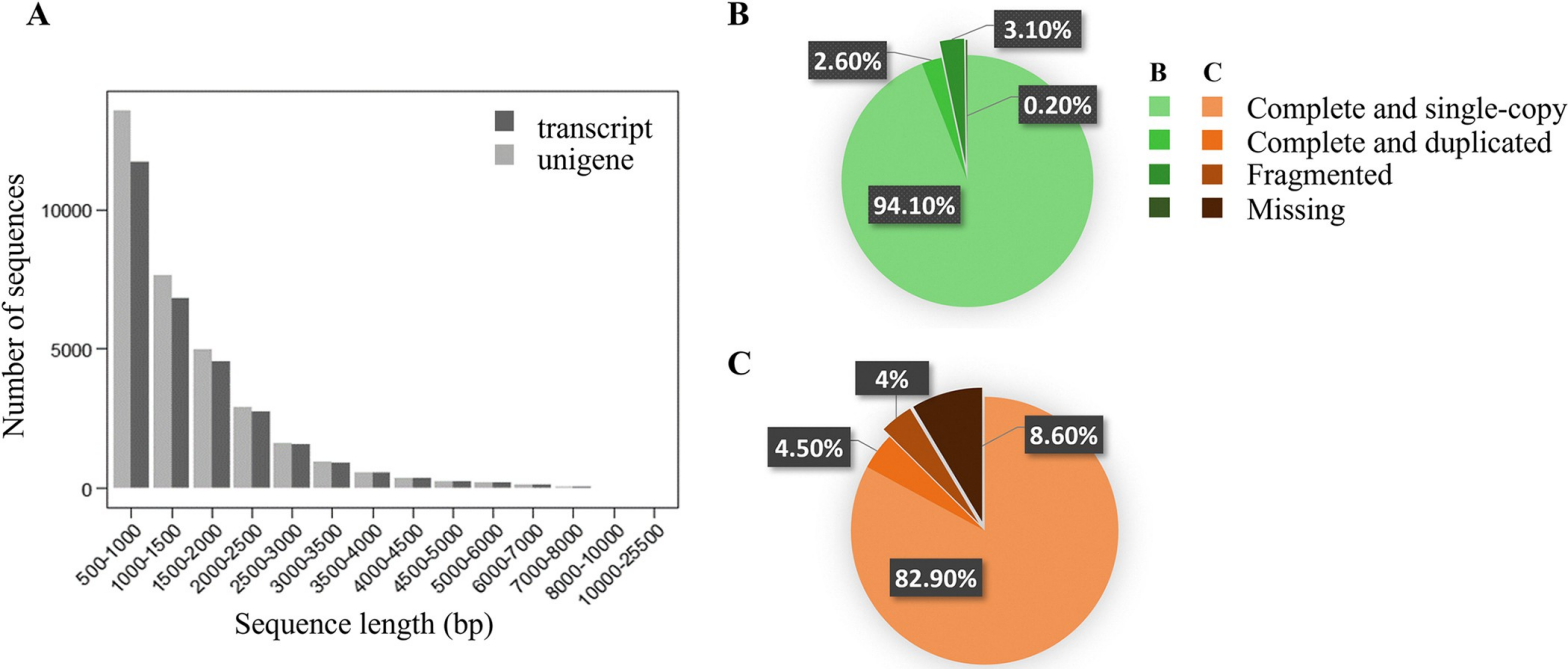
Characteristics and quality assessment of the *de novo* assembly GCAT 4.0. (A) Distribution of the number of transcript and unigene sequences as a function of the sequence length expressed in base pairs (bp). The two pie charts represent the results of the BUSCO analysis using (B) the *viridiplantae* database (Viridiplantae_(odb10) and (C) the *embryophyta* database (Embryophyta_(odb10)).

The *de novo* transcriptome assembly conducted in this study encompasses a total of 33,287 unique transcripts, corresponding to 18,934 unique genes, as determined through BLASTn analysis against the reference genome of white spruce (S2 and S3 Tables). A total of 33,283 potential open reading frames (ORFs) with an average length of 852 base pairs (bp) were identified using the TRAPID pipeline [63]. The contig N50 stands at 1,816 bp, and the contig N90 is 746 bp (S2 Table). Sequence length distribution showed that the transcriptome assembly encompassed a wide range of transcript sizes: 56.2% spanning from 1,000 bp to 4,000 bp, 40.7% ranging from 500 bp to 1,000 bp, and 3.1% exceeding 4,000 bp (Fig 2A), and a median length of 1,173 bp and a mean length of 1,488 bp (S2 Table). Within the 425 *Viridiplantae* odb10 BUSCO groups, 94.1% were identified as complete and single-copy, 2.6% as complete and duplicated, 3.1% as fragmented, and only 0.2% were absent (Fig 2B). Additionally, among the 1,614 *Embryophyta* odb10 BUSCO groups, 82.9% were categorized as complete and single-copy, 4.5% as complete and duplicated, 4.0% as fragmented, and 8.6% were found to be missing (Fig 2C). BLASTx analysis of the *de novo* assembly against public databases revealed sequence homology rates of 65.27% with UniProt, 43.5% with PlantTF, 80.62% with *Viridiplantae* NR, and 79.41% with the NCBI RefSeq database (S4 Table).

### 3.2. Temporal dynamics in gene expression in response to drought

**Fig 3:**
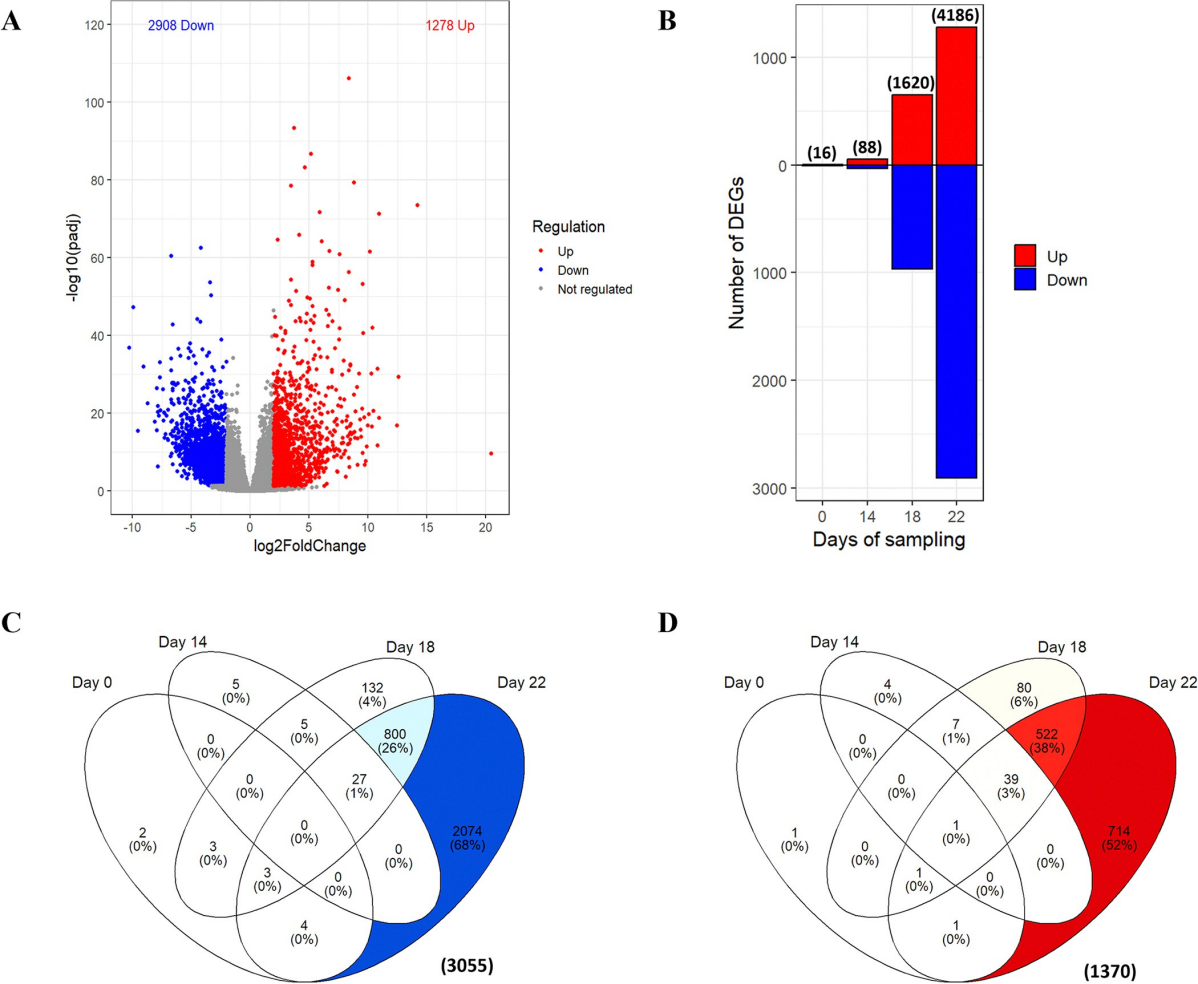
Differentially expressed unigenes (DEGs) in response to drought in white spruce. (A) Volcano plot control versus water stressed white spruce trees on day 22. Down-regulated unigenes (FDR ⩽ 0.05 and a log2FC ⩽ -2) are shown in blue, while up-regulated unigenes (FDR ⩽ 0.05 and a log2FC ⩾ 2) are represented in red. Genes whose expression is not significantly altered by drought are identified by grey dots. (B) The number of differentially expressed unigenes (DEGs) is shown as a function of their regulation (in red, upwards, and in blue, downwards) for the four sampling days. The Venn Diagrams depict the overlaps of (C) downregulated and (D) upregulated differentially expressed genes across the days of sampling. The intensity of the color is positively correlated with the number of unigenes.

Analysis of differentially expressed genes (DEGs) identified 4,425 out of the 18,934 detected unigenes in response to drought, with 1,370 up-regulated unigenes and 3,055 down-regulated unigenes (Fig 3 and S5 Table). As the water stress intensifies over time, an increasing number of both up-regulated and down-regulated genes were observed, showing an initial response affecting a few genes followed by changes in a very large number of genes expressed; 16 DEGs were identified on day 0, followed by 88 genes on day 14. Subsequently, the number of regulated genes escalated to 1,620 on day 18, reaching a substantial peak of 4,186 on day 22 (Fig 3B). The DEGs were not the same from the beginning to the end of the treatment. Specifically, an overlap of 37% was observed exclusively for up-regulated genes between days 18 and 22 (Fig 3D), while a 23% overlap was observed for down-regulated genes (Fig 3C).

### 3.3. Identification of key functions involved in short-term drought response

**Fig 4:**
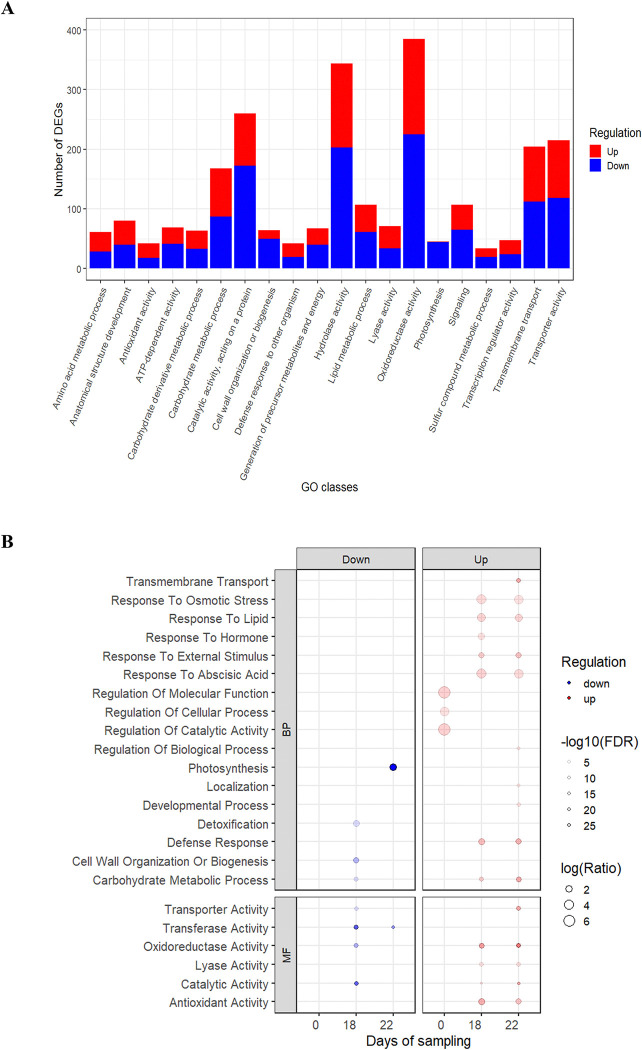
Results of gene ontology enrichment analyses as a function of drought exposure time. (A) The barplot represents the gene ontology (GO) annotation from OmicsBox of unique DEGs regulated on all time points. (B) The scatterplots represent the enriched GO terms belonging to the biological process (BP) and molecular function (MF), as a function of exposure time to the water stress treatment (Days of sampling). Significantly enriched GO terms are shown with the transparency gradient based on -log10(FDR). The size of the dots indicates the ratio of the number of annotated sequences in the sample to the reference transcriptome GCAT 4.0. Enriched GO terms associated with up- and down-regulated sequences are shown in red and blue, respectively. Day 14 showed no significant enrichment and has been withdrawn from the graph for clarity.

The MapMan analysis performed on all sampling days illustrated the key metabolic pathways involved in the water stress response of white spruce seedlings. It showed that 42.8% of the 4,425 drought-responsive genes were assigned to Bins belonging to 29 major functional groups (S6.1 and S6.2 Tables in S6 Table). The most represented functions among unigenes encompassed enzymatic classification (35.76%), solute transport (10.2%), RNA biosynthesis (9.24%), protein modification (6.02%), cell wall organization (4.28%), protein homeostasis (4.44%), phytohormone action (3.54%), photosynthesis (2.8%), carbohydrate metabolism (2.64%), and lipid metabolism (2.59%) (S6.2 Table in S6 Table). Furthermore, the level 3 Gene Ontology (GO) annotation identified highly represented biological processes (BP) such as transmembrane transport (204 DEGs), signaling (107 DEGs), carbohydrate metabolism (168 DEGs), and lipid metabolism (107 DEGs). Notably, both photosynthesis and cell wall biosynthesis/organization processes had a substantial proportion of down-regulated DEGs (95.6 and 78.1%, respectively). The GO annotation also revealed a substantial presence of molecular functions (MF), such as oxidoreductase activity (385 DEGs), hydrolase activity (344 DEGs), and catalytic activity (260 DEGs) (Fig 4A and S7 Table).

**Fig 5:**
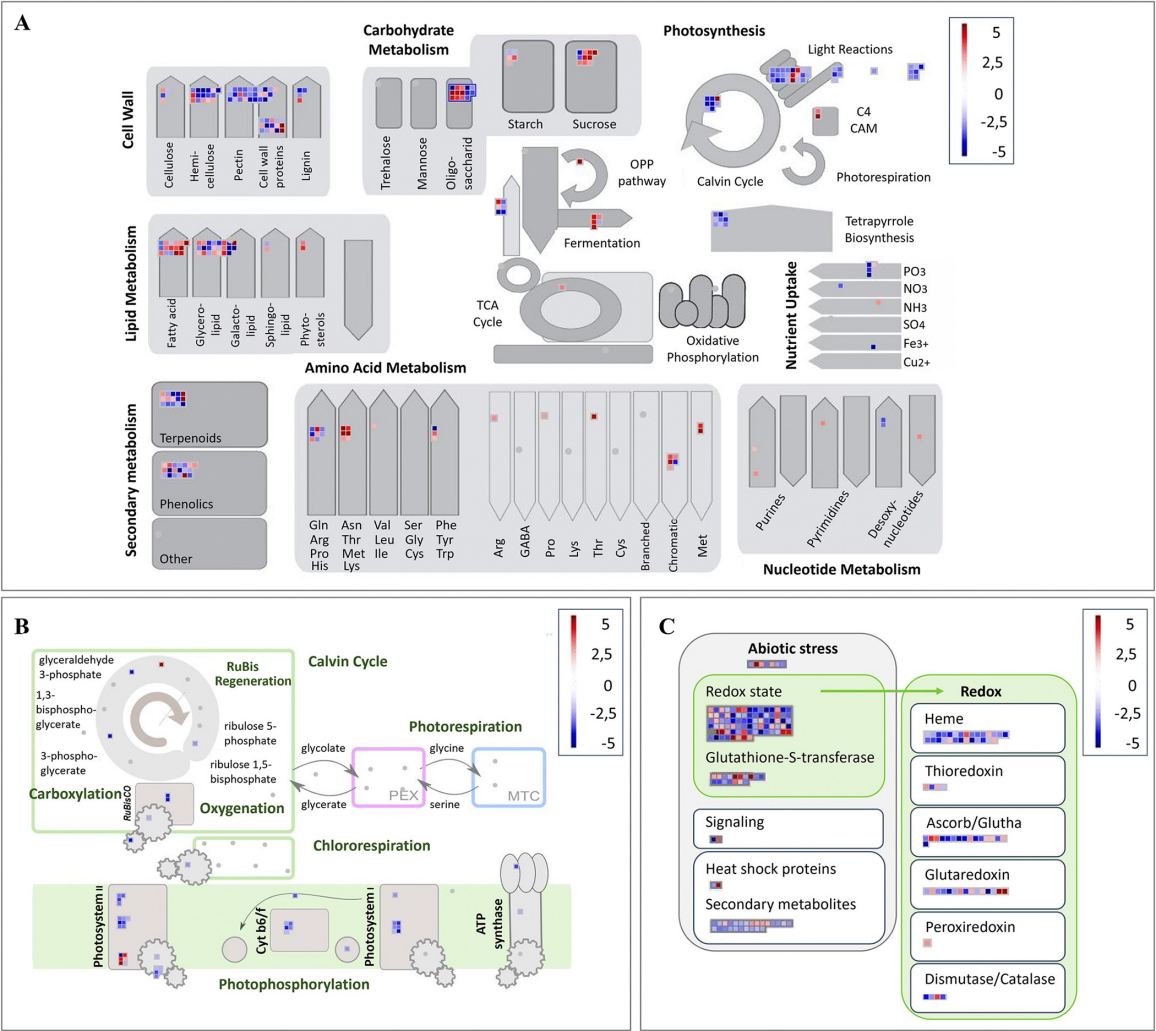
Pathways based on MapMan classification of differentially expressed genes (DEGs) involved in drought stress responses after 22 days in white spruce seedlings. Expression profiles of DEGs involved in metabolism overview (A), photosynthesis (B), abiotic stresses and redox homeostasis (C) are presented. The schematic representation of panel (C) was obtained after modifying MapMan’s original pathways (biotic stress and cellular response overview pathways) to improve and more concisely synthesize the results obtained in the context of our specific short-term drought experiment. The scale bar represents the up- (red) and down- (blue) regulation of gene expression based on log2FC scores.

The GO enrichment analysis performed on differentially expressed transcripts (DETs) indicated that BP and MF changed between the first half of the experiment (0–14 days) and the latter half (18–22 days) in the experiment. Before day 18, MFs associated with the regulation of molecular function, cellular process, and catalytic activity were enriched. In contrast, up-regulated DETs on day 18 and day 22 were associated with responses with osmotic stress, hormone stimuli including abscisic acid (ABA), reactions to external stimuli, defense mechanisms, and carbohydrate and lipid metabolism. The photosynthesis process was among the enriched BPs linked to down-regulated DETs at day 22 (Figs 4A and 4B and 5A and 5B). Several molecular functions were also enriched in both up-regulated and down-regulated DETs, particularly involving catalytic activity and oxidoreductase activity. However, the observed enrichment of lyase and antioxidant activities was unique to up-regulated DETs (Fig 4B, MF panel).

### 3.4. Gene expression profiles at peak water stress: Insights into drought-responsive pathways

A distinct MapMan analysis was conducted only on DEGs at day 22 (Fig 5), which had by far the most water stress responsive DEGs (4,186 DEGs; Fig 3A), and included 68% of the total down-regulated DEGs and 52% of the total up-regulated DEGs (Fig 3C and 3D). The data included DEGs associated with cell wall organization (76 unigenes), with a prevalent down-regulation observed in photosynthesis metabolism (55 unigenes), lipid metabolism (46 unigenes), carbohydrate metabolism (47 unigenes), and secondary metabolism (34 unigenes), primarily connected to terpenoids and phenolic compounds (Fig 5A and 5B). The redox homeostasis process was well-represented with 41 DEGs (Fig 5C and S6.2 and S6.3 Tables in S6 Table). Furthermore, the identification of InterPro domains indicated many DEGs encoding Leucine-rich repeat and kinases proteins, alpha-beta hydrolases, AAA+ ATPases, and cytochrome P450 specifically on day 22. Finally, members of heat shock proteins (HSPs), dehydrins, major intrinsic proteins, and late embryogenesis abundant proteins (LEA) were also detected (S2 Fig).

### 3.5. Identification of drought-responsive transcription factors

**Fig 6:**
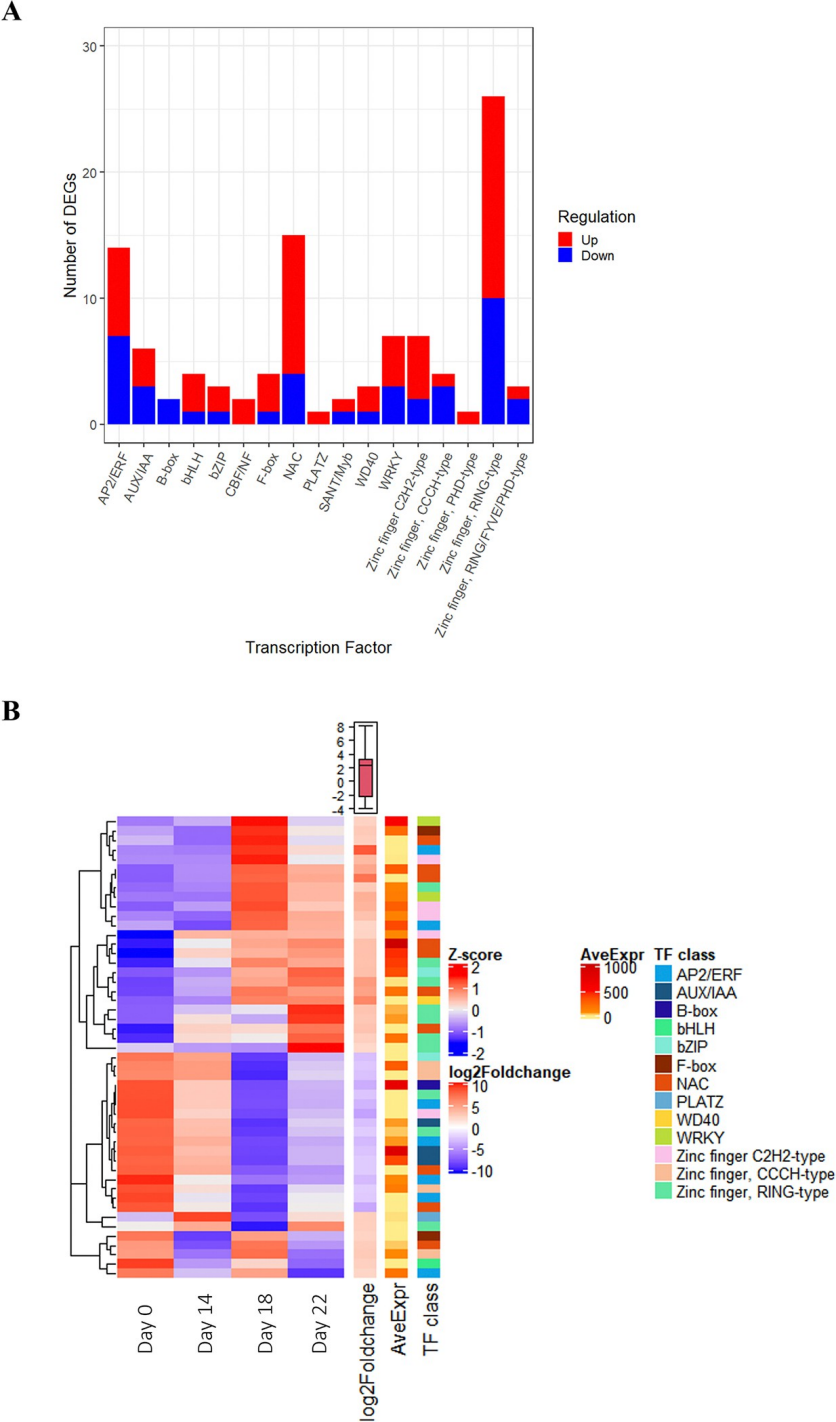
Main classes of transcription factors (TFs) unigenes significantly regulated in response to drought. (A) Histogram showing the number of up (red) or down (blue) regulated genes for the most represented classes of drought-responsive TFs. (B) Heatmap showing the expression of TFs belonging to key TF classes in the response to drought conditions. To the right of the heatmap is the log2 fold change (log2Foldchange), which corresponds to the level of regulation of transcription factor expression when it was detected significantly regulated for a given time point. In cases where a TF was up-regulated at more than one time point, the log2foldchange was averaged over multiple time points and plotted in the heatmap. The complete list of drought-responsive TFs is presented in S8 Table.

A total of 389 potential transcription factors (TFs) were differentially expressed in the present study, 103 of which are considered highly regulated with a LFC greater than 2 or less than -2 with 62 up-regulated and 42 down-regulated unigenes classified into 17 classes. The most represented classes were the RING type zinc fingers (26 DEGs), followed by NAC (15 DEGs) and AP2/ERF (14 DEGs) (Fig 6A, S8.1 and S5.2 Tables in S8 Table). Notably, the RING and C2H2 type zinc finger genes, as well as the WRKY genes, had a predominantly up-regulated expression under drought. The AP2/ERF and AUX/IAA classes contained an equal number of genes with both up and down regulation, while the CBF/NF, PLATZ, and PHD type zinc finger subfamilies exclusively had up-regulated genes. The data showed that most changes in the expression of these TFs occur after 18 days of drought with a notable increase in the magnitude of the LFC (Fig 6B and S8.2 Table in S8 Table). The most up-regulated TFs after 18 days including three NACs, two AP2/ERF, two zinc fingers and one CBF/NF.

### 3.6. Intraspecific genetic variation of drought-responsive genes

The present study used three genetically unrelated clones, and a clone-to-clone analysis identified 638 up-regulated and 63 down-regulated differentially expressed genes, indicating intraspecific gene expression differences under drought (S3 Fig and S9 Table). Overall time points, no down-regulated DEGs were shared among clones (S3B Fig), and only 21% of up-regulated DEGs were common among all three clones (S3A Fig). Shared DEGs were involved in BP of defense mechanisms and macromolecule metabolism covering carbohydrates, lipids, and amino acids (S4A Fig), and were also associated with MF of catalytic activities including transferase, oxidoreductase, lyase, isomerase, and hydrolase activities (S4C Fig). GO enrichment analysis showed that the most enriched BP or MF was similar across all three clones, including catalytic and antioxidant activities, as well as defense response processes (S4 Fig).

## 4. Discussion

This study presents an expanded white spruce transcriptome under water stress, enhancing transcriptomic resources, and characterizing key regulated genes in this conifer model species. The new transcriptome assembly allowed for a great characterization of key genes that are regulated under drought conditions. Our transcriptomic analysis describes the regulation of genes in white spruce after 22 days of water stress, revealing a significant increase in differentially regulated genes (DEGs) compared to controls, with over 4,000 DEGs by day 22. This robust regulation underscores the intensity of the treatment and the strong response in white spruce. The gene expression data suggests that the treatment disrupted numerous physiological processes, as expected for this drought-sensitive species. We identified several drought-responsive genes associated with photosynthesis, growth, water transport, sugar and lipid metabolism, and defense mechanisms.

### 4.1. Quality of the new transcriptome assembly

A new transcriptome assembly of white spruce has been generated based on needles representing different developmental stages (seedlings and saplings) and exposed to various conditions as extreme drought stress. This new assembly complements the previously published and 2011 dated representation of genes expressed under such environmental conditions in white spruce (Fig 1). The completeness achieved in the GCAT 4.0 assembly is consistent with similar investigations conducted on various conifer species [77–80]. Our assembly approach yielded a high proportion of complete and single-copy genes, with minimal redundancy of complete genes (Fig 2). The transcriptome assembly representing roughly 62.26% of the 30,410 estimated genomic gene count [81], highlighting the substantial representation of genes, especially considering that the assembly exclusively originated from needle tissue. The GCAT 4.0 transcriptome assembly represents a robust foundation that complements the previously published assembly to investigate the molecular pathways involved in the response of white spruce needles to drought stress.

### 4.2. Signaling and hormonal response to drought

In response to water deficit, plants initiate a cascade of hormonal and signaling pathways that orchestrate both molecular and physiological responses toward drought tolerance. These processes involve the activation of genes responsible for the synthesis and signaling pathway of the stress hormone abscisic acid (ABA), which is facilitated by a variety of protein kinases and tyrosine phosphatases [82]. Our transcriptomic analysis identified 115 to 385 putative protein kinases with drought-responsive expression with approximately two-thirds being down-regulated. We also observed an up-regulation of three tyrosine phosphatases (S5 Table). Isohydric species, like white spruce, activate early stomatal closure in response to drought [35], with ABA playing a key role in reducing water loss [83, 84]. While it has been traditionally suggested that ABA biosynthesis and signaling occur in the roots before being transported to the leaves to initiate stomatal closure in drought-stressed plants, recent research indicates that these mechanisms may start directly in the leaves of pine and spruce species [85, 86]. In our study, biological processes (BP) related to ABA were enriched on days 18 and 22 (Fig 4B). The differentially expressed genes (DEGs) associated with ABA biosynthesis and signal transduction were primarily identified at days 18 and 22, but some were also detected within the first 14 days of treatment. Specifically, we identified two up-regulated gene related to NCED3 (9-cis-epoxycarotenoid dioxygenase 3), a key enzyme involved in ABA synthesis and previously observed in the drought stress response of *Picea abies* [8] and *Pinus massoniana* [25]. Additionally, we found one up-regulated gene associated with the ABA receptor PYL, which plays a role in inhibiting PP2C (2C-type protein phosphatases), known as a negative regulator of the ABA-signaling enhancer SNF1-related protein kinase (SnRK2) (S5 Table) [8, 25]. Our findings support the significance of ABA-related genes in the response of white spruce and suggest that the intensity and duration of the stress amplify this signaling pathway.

On days 18 and 22, several up-regulated DEGs related to hormones other than ABA, particularly auxin (12 DEGs) and ethylene (6 DEGs) (S5 Table) were observed. We identified three putative up-regulated AUX/IAA sequences, known to be involved in early auxin signaling and regulated in response to drought [87]. We observed five up-regulated and four down-regulated genes belonging to the SAUR (small auxin upregulated RNA) -like auxin-responsive protein family, which may influence tree drought tolerance by establishing leaf auxin concentration gradients and regulating stomatal closure [88]. The expression of five putative Dormancy/auxin-associated proteins, which play pivotal roles in responding to stress and impacting plant growth and development, was also detected (S5 Table) [89]. Our findings indicate the regulation of genes that may play a role in initiating hormonal signal transduction under drought conditions, which is expected to affect growth and photosynthesis in white spruce.

### 4.3. Negative impact of drought on photosynthesis, growth, and water transport

The numerous down-regulated genes linked to photosynthesis, particularly showing a more pronounced decline after 18 and 22 days of drought treatment (Figs 4 and 5B), suggest an abrupt disruption of photosynthesis as the drought stress intensifies. Water availability significantly impacts photosynthesis, often causing a limitation in CO_2_ uptake due to reduced stomatal and mesophyll conductance [90]. Alterations in photosynthesis can also be attributed to metabolic disruptions induced by oxidative stress, leading to the degradation of cellular membranes, components of the electron transport chain, and photosynthetic pigments, among others [91, 92]. Here, three DEGs were associated with rubisco activity, including two encoding Ribulose-1,5-bisphosphate carboxylase/oxygenase and one related to rubisco activase (S5 Table), which plays a pivotal role in the assimilation and fixation of CO_2_ [90]. We observed a down-regulation of genes associated with critical components of the electron transport chain, including one DEG related to the cytochrome b6f complex, seven DEGs associated with Photosystem I (PSI), and four DEGs linked to Photosystem II (PSII). The cytochrome b6f complex expedites the movement of electrons between these two photosystems, resulting in the formation of a proton gradient that drives the synthesis of adenosine triphosphate (ATP) [93]. In plants, PSI and PSII play pivotal roles in capturing light energy and facilitating the transfer of electrons within the electron transport chain [94]. In line with previous studies, our findings suggest that prolonged periods of water stress can adversely affect both PSI and PSII [95, 96]. We also observed a decrease in the expression of 13 DEGs associated with photosynthetic pigments such as chlorophyll a, chlorophyll b, and carotenoids (S5 Table), which is in line with previous research conducted on conifers [92, 96, 97]. Our study highlights a significant disruption of photosynthesis in white spruce under drought conditions, which may be due to both reduced CO_2_ uptake and damage to numerous components within the photosynthetic chain.

Water stress in trees leads to reduced growth, even before a decline in photosynthesis occurs [98, 99], which involves decreased cell wall expansion due to turgor loss and osmotic imbalances, as well as a decline in cell division and wall construction [100]. In our study, two potential osmotin/thaumatin-like (OTL) proteins had decreased expression. These proteins play a role in maintaining cellular osmolarity during stress, as indicated by [101], suggesting a probable osmotic adjustment in white spruce under drought conditions. The regulation of water transport and cell turgor pressure relies on specialized water channels called aquaporins (AQPs). Consistent with the substantial decrease in water potential measured in the same white spruce seedlings subjected to the same drought experiment [6], the down-regulation of ten aquaporins, specifically plasma membrane intrinsic proteins (PIPs), indicated a reduction of water transport in needles. These observations align with previous research in spruces and pines [23, 25, 102] and support a water conservation mechanism by the reduction in AQPs expression during water stress in conifers. The enrichment of down-regulated transcripts related to cell wall organization or biosynthesis (Fig 4A and 4B) highlights the reduction in cell division and wall construction under drought conditions. We identified 19DEGs linked to both the cellulose synthase (CesAs) involved in cellulose synthesis within primary cell walls, and cellulose synthase-like (CSLs) families recognized for their contribution to secondary cell wall synthesis [21]. Consistent with previous findings in water-stressed *Abies alba* seedlings, we observed a decreased expression of genes encoding xyloglucan endotransglucosylase/hydrolase (XTH), a crucial enzyme involved in plant cell wall reconstruction [24, 103]. These findings emphasize the disruption of several crucial growth-related processes in white spruce induced by drought.

### 4.4. Regulation of the carbohydrate and lipid metabolisms

We reported an enrichment of carbohydrate metabolism under drought conditions on days 18 and 22 (Fig 5A). A common defense mechanism in drought-affected trees is to reallocate carbon resources away from growth and toward storage of non-structural carbohydrates (NSCs), such as starch and soluble sugars, e.g., sucrose. The concentration of these compounds increases in root and woody tissues and contributes to maintaining osmotic balance [99, 104]; the compounds may serve as carbon precursors for the synthesis of defense compounds and act as signaling molecules [105]. In our study, five differentially expressed genes (DEGs) were associated with sucrose synthase and eight with the sucrose and hexose transporters SWEETs (S5 Table). This suggests a modulation of sucrose levels in white spruce seedlings under drought conditions, as previously shown in Norway spruce and pine [86, 106]. While competition for a limited pool of available resources has long been considered the driving force behind the trade-off between growth and defense [107], recent findings in *Arabidopsis thaliana* suggest that the incompatibility between growth and defense may also be due to the antagonistic nature of the molecular pathways regulating these two processes [108].

Lipids play essential roles in cell membrane structure, energy storage, and signaling [109]. Various conifer species, such as those found in the *Larix*, *Pinus*, and *Picea* genera, possess substantial lipid reserves [110, 111], but our understanding of lipid metabolism in conifers under water deficit conditions remains limited. Lipid metabolism was altered in response to drought stress in our experiment, primarily affecting glycerolipid metabolism (Fig 5A). Glycerolipids are crucial for thylakoid lipid bilayer formation and efficient photosynthesis, and decreased levels of these molecules have been linked to reduced photosynthesis in higher plants [112]. Drought induced the regulation of genes associated with fatty acid metabolism, leading to the up-regulation of putative malate synthases (3 DEGs), citrate synthases (2 DEGs), and isocitrate lyase (1 DEG) (S5 Table). These enzymes play a crucial role in the glyoxylate cycle, providing essential precursors for gluconeogenesis, the process of converting non-carbohydrate precursors into carbohydrates [113]. While most research on conifers under water stress has traditionally focused on sugar metabolism, lipid metabolism has frequently been underemphasized. Nonetheless, our findings highlight a shift in the regulation of genes associated with lipid metabolism, underscoring its active role in drought responses in white spruce. This aspect merits deeper exploration in coniferous species.

### 4.5. Drought-responsive genes coding for protective defense and stress resistance and resilience

A strong representation of antioxidant activity was observed among the DEGs in our study (Figs 4A and 4B and 5A–5C), with increased expression of putative glutathione peroxidases (3 DEGs), glutathione S-transferases (10 DEGs), peroxidases (10 DEGs) and catalases (3 DEGs) (S5 Table). Many protective molecules such as antioxidants proteins, late embryogenesis abundant proteins (LEA), heat shock proteins (HSPs), and other types of molecules are involved in drought responses of coniferous species [9]. Reactive oxygen species (ROS) can act as signaling molecules initially during stress, but prolonged or intensified stress increases ROS production, disrupting redox balance and causing oxidative stress [114]. Oxidative stress damages various structures and molecules, such as membrane lipids, proteins, photosynthetic pigments, and nucleic acids [115–117]. This damage seems to be avoided by trees through the production of protective enzymes and molecules to maintain homeostasis and counteract oxidative stress. The balance of antioxidant enzymes plays a major role in ROS scavenging mechanism in plants [118, 119], consistent with a role in drought response in conifers [14, 25, 92]. Our study also identified numerous cytochrome P450 genes (CYTs) that were down-regulated (42 DEGs) and up-regulated (7 DEGs) under stress conditions (S5 Table). In contrast, previous observations in *Pinus elliottii* showed only up-regulation of CYTs [120]. CYTs play a crucial role in drought response by contributing to antioxidant activities and defense response in plants [121, 122]. *CsCYT75B1*, a gene of *Citrus sinensis*, was associated with flavonoid metabolism and was highly expressed after drought stress, contributing to drought tolerance by elevating ROS scavenging activities [123]. Due to the interaction of CYTs whose expression is induced with other key genes in response to water stress [122], the pivotal role of CYTs will require further investigation in white spruce and coniferous species.

HSPs and LEA proteins are chaperone proteins that protect cells from abiotic stress by stabilizing proteins and membranes under stress [9, 11]. Drought-responsive genes coding for HSPs (10 DEGs) and Chaperone DnaJ-domain proteins (11 DEGs) were identified in our study. DnaJ proteins are the main co-chaperones modulating the Hsp70 functions [124], and overexpression of the *VaDJI* gene coding for a DnaJ protein conferred ABA insensitivity and drought tolerance in transgenic tobacco [125]. In addition, the expression of 13 LEA genes (S5 Table), including four up-regulated putative dehydrins (S10 Table, GCAT3.3 genes) belonging to the LEA sub-group II [126] was noted. *Pinaceae* dehydrin induction appears to occur after a certain period of drought [11, 127] which could indicate an increasing role of these genes in stress protection as the stress intensity rises. As previously observed in white spruce, we found that the expression of *PgDhn33*, *PgDhn35* and *PgDhn16* was strongly induced, while the expression of *PgDhn37* was repressed (S10 Table). Interestingly, the expression of two key NLRs or NBS-LRRs (nucleotide-binding, leucine-rich-repeat) genes, known to play a central role in plant resilience to stress and linked to resistance pathogens in conifers [128], were induced under drought conditions (GQ03714_K21, GQ03512_J05), as previously reported in white spruce (S10 Table; [129].

### 4.6. Key transcription factors involved in the transcriptional control of drought-responsive genes

Several classes of transcription factors (TFs) including AP2/ERF, NAC, WRKY, MYB, and zinc finger homeodomain TFs were drought-responsive in our study, consistent with other reports in conifer species [14, 15, 25, 130]. However, in *Arabidopsis thaliana*, NAC TFs were reported to be mainly up-regulated in response to drought [131]. Interestingly, a drought-responsive gene annotated as CCCH-type zinc finger (GQ03707_G19) and a WRKY (GQ04107_D16) in our study were also reported as key genes involved in drought adaptation in white spruce [7]. The two MYB sequences identified in this study had homologies with putative *Arabidopsis thaliana* proteins known to enhance protection against oxidative damage or to be involved in growth, phenylpropanoid biosynthesis, and the ABA signaling pathway [132–134]. Drought-induced AP2/ERF genes in our study were close homologs to ethylene responsive elements in other species [135], and to improve drought tolerance in conifers [136]. DREB subfamily genes within the AP2/ERF group, induced in response to drought stress, are known to activate downstream stress resistance genes and enhance plant drought resistance independently of the ABA signaling pathway, as observed in *Arabidopsis thaliana* [137]. Finally, we observed contrasting expression patterns among WRKY members under drought conditions. Similar findings were reported in *Pinus massoniana*, where some WRKY genes responded to drought stress induced by exogenous ABA, resulting in improved drought tolerance in transgenic tobacco plants [138]. Numerous zinc finger TFs were identified in white spruce (S8 Table), and homologs found in the PlantTFDB database indicate a potential role in stomatal aperture, ROS production and drought tolerance [139, 140].

### 4.7. Intraspecific genetic variation in gene regulation under drought stress: Findings and future avenues

Intraspecific genetic variation in drought response is crucial for selection and adaptation in tree populations faced with environmental change [141]. Recent studies in white spruce have highlighted the role of genetic variation among populations [3], as well as the genomic and transcriptomic basis for drought response and resilience [6, 7]. In our study, differences among the three clones in DEGs underlie most of the variance during water stress conditions. Only 21% of the up-regulated and none of the down-regulated genes were common to the three clones, suggesting that the gene network involved in drought response varies widely between genotypes. Alternatively, biological processes and metabolic functions of DEGs were highly similar between genotypes (S9 Table). Dissimilar gene networks and similar metabolic pathways involved in water-stress response among genotypes were also observed for two clones of loblolly pine with opposite phenotypes for drought tolerance [15]. Our results are also congruent with those of the fir *Abies pinsapo* with contrasting gene expression patterns among post-drought phenotypes [130]. Future studies contrasting drought-induced responses between genotypes of various species of conifers and gymnosperms will likely help to appreciate the variance in key metabolic pathways underlying conifer drought responses and improve selection strategies to cope with climate changes. From a prospective standpoint, exploring transcriptome-wide expression within conifer species with diverse ecological preferences holds promise for unraveling the nuanced modulation of gene expression in response to drought. Also, it appears important to investigate responses of epigenetic nature, which are likely to bear an important role in acclimation and adaptation to drought, in addition to modulation of transcriptome-wide expression [11].

## 5. Conclusions

Our study has provided new and valuable transcriptomic data for understanding how white spruce responds to water stress conditions. By conducting a transcriptomic analysis and monitoring white spruce over time during water stress, we have identified specific gene sets at different time points (days 0, 14, 18 and 22) that shed light on the response to drought. The genes we identified are involved in major known biological processes, including hormonal responses, photosynthesis, growth, cell wall organization, water transport, carbohydrate metabolism, and defense mechanisms, all of which are essential for drought tolerance and acclimation. Our results at the transcriptome level confirm the hypothesis that white spruce seedlings appear to prioritize maintaining their hydraulic balance over growth during a short drought. Furthermore, the results also highlight the ability of white spruce seedlings to activate defense mechanisms, such as antioxidant defenses and chaperone proteins, under such stress conditions. To further understand the drought response of this species, it is crucial to validate these transcriptomic observations using other omics approaches, such as proteomics and metabolomics. One particularly interesting finding is the significant regulation of lipid metabolism, a process that has not been extensively studied in conifers and requires further investigation. Given the role of lipids as energy reserves and precursors of defence compounds, future studies should focus on elucidating their contribution to tree responses to drought, by targeting the key molecular pathways involved during water stress through enzyme activity measurements and lipid profiling. We believe that future research should prioritize the identification and the comparison of key genes and mechanisms involved in drought response and post-drought recovery of plants. This information could be instrumental in shaping effective genetic selection strategies to enhance white spruce’s resistance and resilience in the face of drought induced by climate change. Ultimately, this knowledge can inform forest management practices aimed at supporting conifer regeneration and growth in increasingly challenging dry conditions.

## Supporting information

S1 Fig Volcano plots of differentially expressed genes (DEGs). Volcano plots of DEGs are presented for days 0, 14, 18 and 22 (A-D). Unigenes in blue are under-represented in stressed seedlings (FDR ⩽ 0.05 and a log2FC ⩽ -2), while transcripts in red are over-represented in stressed seedlings (FDR ⩽ 0.05 and a log2FC ⩾ 2). Unigenes in gray are not significantly different between the two groups.
(TIF)

S2 Fig Major protein families involved in the drought response among the differentially expressed genes (DEGs) at day 22. The plot represents the number of up- and down-regulated DEGs at day 22 encoding selected protein families with known roles in plant drought response.
(TIF)

S3 Fig Unique and shared differentially expressed genes (DEGs) among clones. The Venn diagrams depict the overlaps of (A) up-regulated (red) and (B) down-regulated (blue) DEGs across the C8, C11, and C95 clones. The total number of DEGs are shown in bold brackets.
(TIF)

S4 Fig Gene Ontology (GO) annotation of unique and shared differentially expressed genes (DEGs) among clones. GO annotation of biological process (BP) of (A) up- and (B) down-regulated DEGs of clones and GO annotation of molecular functions (MF) of (C) up- and (D) down-regulated DEGs. Unique DEGs correspond to C8, C11 or C95 and the shared DEGs among clones correspond to C8-C11, C11-C95 and C8-C11-C95.
(TIF)

S1 Table Summary of plant material used for de novo transcriptome assembly and transcriptomic analyses. (XLSX)

S2 Table Statistics summary of the *de novo* transcriptome assembly GCAT 4.0. (XLSX)

S3 Table Complete functional annotation of the *de novo* transcriptome assembly GCAT 4.0 based on the OmicsBox analysis. The Excel file contains four sheets; Caption, S3.1 (Blast2GO annotation and BLASTX), S3.2 (BLASTN WS77111v2), S3.3 (BLASTN GCAT3.3).
(XLSX)

S4 Table Summary of BLASTX results of the *de novo* transcriptome assembly GCAT 4.0. The table gathers the results of BLASTX expressed in percentage realized with different public databases. The refSeq_29sep22 database (NCBI) was used in Blast2GO pro suite for our analyses. The meaning of variables: DB: database; DB sequences (%): the percentage of matched sequence; DB unique sequences (%): the percentage of unique matched sequence; Unigenes (%): the percentage of matched unigene; Unique unigenes (%): the percentage of unique matched unigenes. The genome used is the reference genome of white spruce WS77111v2 (https://www.ncbi.nlm.nih.gov/datasets/genome/GCA_000966675.3).
(XLSX)

S5 Table List of differentially expressed transcripts (DETs) as a function of time points and their corresponding genes (DEGs) under drought. The Excel file contains two sheets: Caption and S5 (results of DEG analysis and annotation according to time points).
(XLSX)

S6 Table MapMan analyses performed on differentially expressed genes (DEGs) from all sampling days and on DEGs from day 22. The Excel file contains five sheets; Caption, S6.1 (export results of all sampling days), S6.2 (the main Bin categories of all sampling days), S6.3 (export results of day 22) and S6.4 (the main Bin categories of day 22).
(XLSX)

S7 Table Gene Ontology (GO) enrichment of differentially expressed transcripts (DETs) as a function of time points under drought. The Excel file contains three sheets; Caption, S7.1 (GO enrichment according to time points) and S7.2 (selected GO enrichment according to time points).
(XLSX)

S8 Table Transcription Factors (TFs) annotation of differentially expressed transcripts (DETs) and their corresponding genes (DEGs). The Excel file contains three sheets; Caption, S5.1 (TFs annotation among all transcripts) and S5.2 (TFs annotation among selected DEGs).
(XLSX)

S9 Table List of differentially expressed transcripts (DETs) and Gene Ontology (GO) enrichment as a function of clones and their corresponding genes (DEGs) under drought. The Excel file contains three sheets; Caption, S7.1 (results of DEG analysis and annotation according to clones) and S7.2 (GO enrichment according to clones).
(XLSX)

S10 Table Cross-checking information from previously published studies on differentially expressed transcripts (DETs) and their corresponding genes (DEGs). The Excel file contains two sheets; Caption and S10 (list of key drought-responsive genes previously observed in published studies).
(XLSX)

S1 Methods Methodological details of experiences used for *de novo* transcriptome assembly. (DOCX)

## References

[1] Forzieri G, Dakos V, McDowell NG, Ramdane A, Cescatti A. Emerging signals of declining forest resilience under climate change. Nature. 2022;608: 534–539. doi: 10.1038/s41586-022-04959-9 35831499 PMC9385496

[2] Hartmann H, Bastos A, Das AJ, Esquivel-Muelbert A, Hammond WM, Martínez-Vilalta J, et al. Climate change risks to global forest health: emergence of unexpected events of elevated tree mortality worldwide. Annu Rev Plant Biol. 2022;73: 673–702. doi: 10.1146/annurev-arplant-102820-012804 35231182

[3] Depardieu C, Girardin MP, Nadeau S, Lenz P, Bousquet J, Isabel N. Adaptive genetic variation to drought in a widely distributed conifer suggests a potential for increasing forest resilience in a drying climate. New Phytol. 2020;227: 427–439. doi: 10.1111/nph.16551 32173867 PMC7317761

[4] Laverdière J, Lenz P, Nadeau S, Depardieu C, Isabel N, Perron M, et al. Breeding for adaptation to climate change: genomic selection for drought response in a white spruce multi‐site polycross test. Evol Appl. 2022;15: 383–402. doi: 10.1111/eva.13348 35386396 PMC8965362

[5] Soro A, Lenz P, Roussel J-R, Larochelle F, Bousquet J, Achim A. The phenotypic and genetic effects of drought-induced stress on apical growth, ring width, wood density and biomass in white spruce seedlings. New For. 2023;54: 789–811. doi: 10.1007/s11056-022-09939-5 PMC10766765 38186604

[6] Stival Sena J, Giguère I, Rigault P, Bousquet J, Mackay J. Expansion of the dehydrin gene family in the *Pinaceae* is associated with considerable structural diversity and drought-responsive expression. Tree Physiol. 2018;38: 442–456. doi: 10.1093/treephys/tpx125 29040752

[7] Depardieu C, Gérardi S, Nadeau S, Parent GJ, Mackay J, Lenz P, et al. Connecting tree-ring phenotypes, genetic associations and transcriptomics to decipher the genomic architecture of drought adaptation in a widespread conifer. Mol Ecol. 2021;30: 3898–3917. doi: 10.1111/mec.15846 33586257 PMC8451828

[8] Haas JC, Vergara A, Serrano AR, Mishra S, Hurry V, Street NR. Candidate regulators and target genes of drought stress in needles and roots of Norway spruce. Plomion C, editor. Tree Physiol. 2021;41: 1230–1246. doi: 10.1093/treephys/tpaa178 33416078 PMC8271197

[9] Baldi P, La Porta N. Toward the genetic improvement of drought tolerance in conifers: an integrated approach. Forests. 2022;13: 2016. doi: 10.3390/f13122016

[10] Hamanishi ET, Campbell MM. Genome-wide responses to drought in forest trees. For Int J For Res. 2011;84: 273–283. doi: 10.1093/forestry/cpr012

[11] Moran E, Lauder J, Musser C, Stathos A, Shu M. The genetics of drought tolerance in conifers. New Phytol. 2017;216: 1034–1048. doi: 10.1111/nph.14774 28895167

[12] Wang Y, Zhao Z, Liu F, Sun L, Hao F. Versatile roles of aquaporins in plant growth and development. Int J Mol Sci. 2020;21: 9485. doi: 10.3390/ijms21249485 33322217 PMC7763978

[13] Laoué J, Depardieu C, Gérardi S, Lamothe M, Bomal C, Azaiez A, et al. Combining QTL mapping and transcriptomics to decipher the genetic architecture of phenolic compounds metabolism in the conifer white spruce. Front Plant Sci. 2021;12: 675108. doi: 10.3389/fpls.2021.675108 34079574 PMC8166253

[14] Fox H, Doron-Faigenboim A, Kelly G, Bourstein R, Attia Z, Zhou J, et al. Transcriptome analysis of *Pinus halepensis* under drought stress and during recovery. Tree Physiol. 2018;38: 423–441. doi: 10.1093/treephys/tpx137 29177514 PMC5982726

[15] Li W, Lee J, Yu S, Wang F, Lv W, Zhang X, et al. Characterization and analysis of the transcriptome response to drought in *Larix kaempferi* using PacBio full-length cDNA sequencing integrated with *de novo* RNA-seq reads. Planta. 2021;253: 28. doi: 10.1007/s00425-020-03555-3 33423138

[16] He W, Liu H, Qi Y, Liu F, Zhu X. Patterns in nonstructural carbohydrate contents at the tree organ level in response to drought duration. Glob Change Biol. 2020;26: 3627–3638. doi: 10.1111/gcb.15078 32162388

[17] Zhang L, Yan S, Zhang S, Yan P, Wang J, Zhang H. Glutathione, carbohydrate and other metabolites of *Larix olgensis* A. Henry reponse to polyethylene glycol-simulated drought stress. PLOS ONE. 2021;16: e0253780. doi: 10.1371/journal.pone.0253780 34788320 PMC8598043

[18] Sancho-Knapik D, Sanz MÁ, Peguero-Pina JJ, Niinemets Ü, Gil-Pelegrín E. Changes of secondary metabolites in *Pinus sylvestris* L. needles under increasing soil water deficit. Ann For Sci. 2017;74: 24. doi: 10.1007/s13595-017-0620-7

[19] Xiao F, Zhao Y, Wang X-R, Liu Q, Ran J. Transcriptome analysis of needle and root of Pinus massoniana in response to continuous drought stress. Plants. 2021;10: 769. doi: 10.3390/plants10040769 33919844 PMC8070838

[20] Velasco-Conde T, Yakovlev I, Majada J, Aranda I, Johnsen Ø. Dehydrins in maritime pine (*Pinus pinaster*) and their expression related to drought stress response. Tree Genet Genomes. 2012;8: 957–973. doi: 10.1007/s11295-012-0476-9

[21] Wang T, McFarlane HE, Persson S. The impact of abiotic factors on cellulose synthesis. J Exp Bot. 2016;67: 543–552. doi: 10.1093/jxb/erv488 26552883

[22] Coleman HD, Brunner AM, Tsai C-J. Synergies and entanglement in secondary cell wall development and abiotic stress response in trees. Front Plant Sci. 2021;12. doi: 10.3389/fpls.2021.639769 33815447 PMC8018706

[23] Lorenz WW, Alba R, Yu Y-S, Bordeaux JM, Simões M, Dean JF. Microarray analysis and scale-free gene networks identify candidate regulators in drought-stressed roots of loblolly pine (*P*. *taeda L*.). BMC Genomics. 2011;12: 264. doi: 10.1186/1471-2164-12-264 21609476 PMC3123330

[24] Behringer D, Zimmermann H, Ziegenhagen B, Liepelt S. Differential gene expression reveals candidate genes for drought stress response in *Abies alba* (*Pinaceae*). Prasad M, editor. PLoS ONE. 2015;10: e0124564. doi: 10.1371/journal.pone.0124564 25924061 PMC4414588

[25] Du M, Ding G, Cai Q. The transcriptomic responses of *Pinus massoniana* to drought stress. Forests. 2018;9: 326. doi: 10.3390/f9060326

[26] Zhang S, Koubaa A. Softwoods of eastern Canada: Their silvics, characteristics, manufacturing and end-uses. Special Publication SP-526E. Forintek Canada Corporation; 2008.

[27] Hassegawa M, Savard M, Lenz PRN, Duchateau E, Gélinas N, Bousquet J, et al. White spruce wood quality for lumber products: priority traits and their enhancement through tree improvement. For Int J For Res. 2020;93: 16–37. doi: 10.1093/forestry/cpz050

[28] Mullin T, Andersson Gull B, Bastien J-C, Beaulieu J, Burdon R, Dvorak W, et al. Economic importance, breeding objectives and achievements. Plomion, C., and Bousquet J. Genetics, Genomics and Breeding of Conifers. Plomion, C., and Bousquet J. CRC Press and Science Publishers, New York; 2011. pp. 40–127. doi: 10.1201/b11075-3

[29] Bousquet J, Gérardi S, De Lafontaine G, Jaramillo-Correa JP, Pavy N, Prunier J, et al. Spruce population genomics. Rajora, O.P. Population Genomics: Forest Trees. Rajora O.P. Springer Nature, Switzerland; 2021. pp. 1–64. doi: 10.1007/13836_2021_96

[30] Hogg EH, Michaelian M, Hook TI, Undershultz ME. Recent climatic drying leads to age-independent growth reductions of white spruce stands in western Canada. Glob Change Biol. 2017;23: 5297–5308. doi: 10.1111/gcb.13795 28636146

[31] Sullivan PF, Brownlee AH, Ellison SBZ, Cahoon SMP. Comparative drought sensitivity of co‐occurring white spruce and paper birch in interior Alaska. Bellingham P, editor. J Ecol. 2021;109: 2448–2460. doi: 10.1111/1365-2745.13654

[32] Peng C, Ma Z, Lei X, Zhu Q, Chen H, Wang W, et al. A drought-induced pervasive increase in tree mortality across Canada’s boreal forests. Nat Clim Change. 2011;1: 467–471. doi: 10.1038/nclimate1293

[33] Lu P, Parker WC, Colombo SJ, Skeates DA. Temperature-induced growing season drought threatens survival and height growth of white spruce in southern Ontario, Canada. For Ecol Manag. 2019;448: 355–363. doi: 10.1016/j.foreco.2019.06.022

[34] D’Orangeville L, Houle D, Duchesne L, Phillips RP, Bergeron Y, Kneeshaw D. Beneficial effects of climate warming on boreal tree growth may be transitory. Nat Commun. 2018;9: 3213. doi: 10.1038/s41467-018-05705-4 30097584 PMC6086880

[35] Brodribb TJ, McAdam SAM, Jordan GJ, Martins SCV. Conifer species adapt to low-rainfall climates by following one of two divergent pathways. Proc Natl Acad Sci U S A. 2014;111: 14489–14493. doi: 10.1073/pnas.1407930111 25246559 PMC4210017

[36] McDowell N, Pockman WT, Allen CD, Breshears DD, Cobb N, Kolb T, et al. Mechanisms of plant survival and mortality during drought: why do some plants survive while others succumb to drought? New Phytol. 2008;178: 719–739. doi: 10.1111/j.1469-8137.2008.02436.x 18422905

[37] Depardieu C, Lenz P, Marion J, Nadeau S, Girardin MP, Marchand W, et al. Contrasting physiological strategies explain heterogeneous responses to severe drought conditions within local populations of a widespread conifer. Sci Total Environ. 2024; 171174. doi: 10.1016/j.scitotenv.2024.171174 38402972

[38] Gazol A, Fajardo A, Camarero JJ. Contributions of intraspecific variation to drought tolerance in trees. Curr For Rep. 2023 [cited 9 Oct 2023]. doi: 10.1007/s40725-023-00199-w

[39] Hornoy B, Pavy N, Gérardi S, Beaulieu J, Bousquet J. Genetic adaptation to climate in white spruce involves small to moderate allele frequency shifts in functionally diverse genes. Genome Biol Evol. 2015;7: 3269–3285. doi: 10.1093/gbe/evv218 26560341 PMC4700950

[40] Birol I, Raymond A, Jackman SD, Pleasance S, Coope R, Taylor GA, et al. Assembling the 20 Gb white spruce (*Picea glauca*) genome from whole-genome shotgun sequencing data. Bioinformatics. 2013;29: 1492–1497. doi: 10.1093/bioinformatics/btt178 23698863 PMC3673215

[41] Warren RL, Keeling CI, Yuen MMS, Raymond A, Taylor GA, Vandervalk BP, et al. Improved white spruce (*Picea glauca*) genome assemblies and annotation of large gene families of conifer terpenoid and phenolic defense metabolism. Plant J. 2015;83: 189–212. doi: 10.1111/tpj.12886 26017574

[42] Jackman SD, Warren RL, Gibb EA, Vandervalk BP, Mohamadi H, Chu J, et al. Organellar genomes of white spruce (*Picea glauca*): assembly and annotation. Genome Biol Evol. 2016;8: 29–41. doi: 10.1093/gbe/evv244 26645680 PMC4758241

[43] Rigault P, Boyle B, Lepage P, Cooke JEK, Bousquet J, MacKay JJ. A white spruce gene catalog for conifer genome analyses. Plant Physiol. 2011;157: 14–28. doi: 10.1104/pp.111.179663 21730200 PMC3165865

[44] Pavy N, Pelgas B, Beauseigle S, Blais S, Gagnon F, Gosselin I, et al. Enhancing genetic mapping of complex genomes through the design of highly-multiplexed SNP arrays: application to the large and unsequenced genomes of white spruce and black spruce. BMC Genomics. 2008;9: 21. doi: 10.1186/1471-2164-9-21 18205909 PMC2246113

[45] Pavy N, Gagnon F, Rigault P, Blais S, Deschênes A, Boyle B, et al. Development of high-density SNP genotyping arrays for white spruce (*Picea glauca*) and transferability to subtropical and nordic congeners. Mol Ecol Resour. 2013;13: 324–336. doi: 10.1111/1755-0998.12062 23351128

[46] Pavy N, Gagnon F, Deschênes A, Boyle B, Beaulieu J, Bousquet J. Development of highly reliable *in silico* SNP resource and genotyping assay from exome capture and sequencing: an example from black spruce (*Picea mariana*). Mol Ecol Resour. 2016;16: 588–598. doi: 10.1111/1755-0998.12468 26391535

[47] Pelgas B, Bousquet J, Meirmans PG, Ritland K, Isabel N. QTL mapping in white spruce: gene maps and genomic regions underlying adaptive traits across pedigrees, years and environments. BMC Genomics. 2011;12: 145. doi: 10.1186/1471-2164-12-145 21392393 PMC3068112

[48] Pavy N, Lamothe M, Pelgas B, Gagnon F, Birol I, Bohlmann J, et al. A high-resolution reference genetic map positioning 8.8 K genes for the conifer white spruce: structural genomics implications and correspondence with physical distance. Plant J. 2017;90: 189–203. doi: 10.1111/tpj.13478 28090692

[49] De La Torre AR, Birol I, Bousquet J, Ingvarsson P, Jansson S, Jones S, et al. Insights into conifer giga-genomes. Plant Physiol. 2014;166. doi: 10.1104/pp.114.248708 25349325 PMC4256843

[50] Neale DB, Wheeler NC. Gene expression and the transcriptome. The conifers: genomes, variation and evolution. Cham: Springer International Publishing; 2019. pp. 91–117. doi: 10.1007/978-3-319-46807-5_6

[51] Jaramillo-Correa JP, Beaulieu J, Bousquet J. Contrasting evolutionary forces driving population structure at expressed sequence tag polymorphisms, allozymes and quantitative traits in white spruce. Mol Ecol. 2001;10: 2729–2740. doi: 10.1046/j.0962-1083.2001.01386.x 11883886

[52] Prunier J, Laroche J, Beaulieu J, Bousquet J. Scanning the genome for gene SNPs related to climate adaptation and estimating selection at the molecular level in boreal black spruce. Mol Ecol. 2011;20: 1702–1716. doi: 10.1111/j.1365-294X.2011.05045.x 21375634

[53] Fujita M, Fujita Y, Noutoshi Y, Takahashi F, Narusaka Y, Yamaguchi-Shinozaki K, et al. Crosstalk between abiotic and biotic stress responses: a current view from the points of convergence in the stress signaling networks. Curr Opin Plant Biol. 2006;9: 436–442. doi: 10.1016/j.pbi.2006.05.014 16759898

[54] Leisner CP, Potnis N, Sanz-Saez A. Crosstalk and trade-offs: plant responses to climate change-associated abiotic and biotic stresses. Plant Cell Environ. 2023;46: 2946–2963. doi: 10.1111/pce.14532 36585762

[55] Andrews S. FastQC: a quality control tool for high throughput sequence data. 2017.

[56] Bolger AM, Lohse M, Usadel B. Trimmomatic: a flexible trimmer for Illumina sequence data. Bioinformatics. 2014;30: 2114–2120. doi: 10.1093/bioinformatics/btu170 24695404 PMC4103590

[57] Coil D, Jospin G, Darling AE. A5-miseq: an updated pipeline to assemble microbial genomes from Illumina MiSeq data. Bioinforma Oxf Engl. 2015;31: 587–589. doi: 10.1093/bioinformatics/btu661 25338718

[58] Simpson JT, Durbin R. Efficient *de novo* assembly of large genomes using compressed data structures. Genome Res. 2012;22: 549–556. doi: 10.1101/gr.126953.111 22156294 PMC3290790

[59] Peng Y, Leung HCM, Yiu SM, Chin FYL. IDBA-UD: A *de novo* assembler for single-cell and metagenomic sequencing data with highly uneven depth. Bioinformatics. 2012;28: 1420–1428. doi: 10.1093/bioinformatics/bts174 22495754

[60] Boetzer M, Henkel CV, Jansen HJ, Butler D, Pirovano W. Scaffolding pre-assembled contigs using SSPACE. Bioinformatics. 2011;27: 578–579. doi: 10.1093/bioinformatics/btq683 21149342

[61] Warren RL, Yang C, Vandervalk BP, Behsaz B, Lagman A, Jones SJM, et al. LINKS: Scalable, alignment-free scaffolding of draft genomes with long reads. GigaScience. 2015;4: 35. doi: 10.1186/s13742-015-0076-3 26244089 PMC4524009

[62] Manni M, Berkeley MR, Seppey M, Zdobnov EM. Busco: assessing genomic data quality and beyond. Curr Protoc. 2021;1: e323. doi: 10.1002/cpz1.323 34936221

[63] Bucchini F, Del Cortona A, Kreft Ł, Botzki A, Van Bel M, Vandepoele K. TRAPID 2.0: a web application for taxonomic and functional analysis of *de novo* transcriptomes. Nucleic Acids Res. 2021;49: e101–e101. doi: 10.1093/nar/gkab565 34197621 PMC8464036

[64] Götz S, García-Gómez JM, Terol J, Williams TD, Nagaraj SH, Nueda MJ, et al. High-throughput functional annotation and data mining with the Blast2GO suite. Nucleic Acids Res. 2008;36: 3420–3435. doi: 10.1093/nar/gkn176 18445632 PMC2425479

[65] Conesa A, Götz S, García-Gómez JM, Terol J, Talón M, Robles M. Blast2GO: a universal tool for annotation, visualization and analysis in functional genomics research. Bioinforma Oxf Engl. 2005;21: 3674–3676. doi: 10.1093/bioinformatics/bti610 16081474

[66] Buchfink B, Reuter K, Drost H-G. Sensitive protein alignments at tree-of-life scale using DIAMOND. Nat Methods. 2021;18: 366–368. doi: 10.1038/s41592-021-01101-x 33828273 PMC8026399

[67] Van Bel M, Silvestri F, Weitz EM, Kreft L, Botzki A, Coppens F, et al. PLAZA 5.0: extending the scope and power of comparative and functional genomics in plants. Nucleic Acids Res. 2022;50: D1468–D1474. doi: 10.1093/nar/gkab1024 34747486 PMC8728282

[68] Jin J, Tian F, Yang D-C, Meng Y-Q, Kong L, Luo J, et al. PlantTFDB 4.0: toward a central hub for transcription factors and regulatory interactions in plants. Nucleic Acids Res. 2017;45: D1040–D1045. doi: 10.1093/nar/gkw982 27924042 PMC5210657

[69] Tian F, Yang D-C, Meng Y-Q, Jin J, Gao G. PlantRegMap: charting functional regulatory maps in plants. Nucleic Acids Res. 2020;48: D1104–D1113. doi: 10.1093/nar/gkz1020 31701126 PMC7145545

[70] Bray NL, Pimentel H, Melsted P, Pachter L. Near-optimal probabilistic RNA-seq quantification. Nat Biotechnol. 2016;34: 525–527. doi: 10.1038/nbt.3519 27043002

[71] Love MI, Huber W, Anders S. Moderated estimation of fold change and dispersion for RNA-seq data with DESeq2. Genome Biol. 2014;15: 550. doi: 10.1186/s13059-014-0550-8 25516281 PMC4302049

[72] Gao C-H, Yu G, Cai P. ggVennDiagram: an intuitive, easy-to-use, and highly customizable R package to generate Venn diagram. Front Genet. 2021;12: 706907. doi: 10.3389/fgene.2021.706907 34557218 PMC8452859

[73] Guo K, McGregor B. VennDetail: A package for visualization and extract details. 2024. Available: https://github.com/guokai8/VennDetail.

[74] Thimm O, Bläsing O, Gibon Y, Nagel A, Meyer S, Krüger P, et al. MAPMAN: a user-driven tool to display genomics data sets onto diagrams of metabolic pathways and other biological processes. Plant J Cell Mol Biol. 2004;37: 914–939. doi: 10.1111/j.1365-313x.2004.02016.x 14996223

[75] Schwacke R, Ponce-Soto GY, Krause K, Bolger AM, Arsova B, Hallab A, et al. Mapman4: a refined protein classification and annotation framework applicable to multi-omics data analysis. Mol Plant. 2019;12: 879–892. doi: 10.1016/j.molp.2019.01.003 30639314

[76] Bolger M, Schwacke R, Usadel B. Mapman visualization of rna-seq data using mercator4 functional annotations. Methods Mol Biol Clifton NJ. 2021;2354: 195–212. doi: 10.1007/978-1-0716-1609-3_9 34448161

[77] Lee IH, Han H, Koh YH, Kim IS, Lee S-W, Shim D. Comparative transcriptome analysis of Pinus densiflora following inoculation with pathogenic (Bursaphelenchus xylophilus) or non-pathogenic nematodes (B. thailandae). Sci Rep. 2019;9: 12180. doi: 10.1038/s41598-019-48660-w 31434977 PMC6704138

[78] Ojeda DI, Mattila TM, Ruttink T, Kujala ST, Kärkkäinen K, Verta J-P, et al. Utilization of tissue ploidy level variation in de novo transcriptome assembly of Pinus sylvestris. G3 GenesGenomesGenetics. 2019;9: 3409–3421. doi: 10.1534/g3.119.400357 31427456 PMC6778806

[79] Breidenbach N, Sharov VV, Gailing O, Krutovsky KV. De novo transcriptome assembly of cold stressed clones of the hexaploid Sequoia sempervirens (D. Don) Endl. Sci Data. 2020;7: 239. doi: 10.1038/s41597-020-00576-1 32681057 PMC7367877

[80] Visser EA, Kampmann TP, Wegrzyn JL, Naidoo S. Multispecies comparison of host responses to *Fusarium circinatum* challenge in tropical pines show consistency in resistance mechanisms. Plant Cell Environ. 2023;46: 1705–1725. doi: 10.1111/pce.14522 36541367

[81] Gagalova KK, Warren RL, Coombe L, Wong J, Nip KM, Yuen MMS, et al. Spruce giga-genomes: structurally similar yet distinctive with differentially expanding gene families and rapidly evolving genes. Plant J. 2022;111: 1469–1485. doi: 10.1111/tpj.15889 35789009

[82] Klápště J, Dungey HS, Telfer EJ, Suontama M, Graham NJ, Li Y, et al. Marker selection in multivariate genomic prediction improves accuracy of low heritability traits. Front Genet. 2020;11. doi: 10.3389/fgene.2020.499094 33193595 PMC7662070

[83] Brodribb TJ, McAdam SAM. Abscisic acid mediates a divergence in the drought response of two conifers. Plant Physiol. 2013;162: 1370–1377. doi: 10.1104/pp.113.217877 23709665 PMC3707560

[84] Brunner I, Herzog C, Dawes MA, Arend M, Sperisen C. How tree roots respond to drought. Front Plant Sci. 2015;6. doi: 10.3389/fpls.2015.00547 26284083 PMC4518277

[85] Mitchell PJ, McAdam SAM, Pinkard EA, Brodribb TJ. Significant contribution from foliage-derived ABA in regulating gas exchange in *Pinus radiata*. Tree Physiol. 2017;37: 236–245. doi: 10.1093/treephys/tpw092 28399262

[86] Pashkovskiy PP, Vankova R, Zlobin IE, Dobrev P, Ivanov YV, Kartashov AV, et al. Comparative analysis of abscisic acid levels and expression of abscisic acid-related genes in Scots pine and Norway spruce seedlings under water deficit. Plant Physiol Biochem. 2019;140: 105–112. doi: 10.1016/j.plaphy.2019.04.037 31091491

[87] Luo J, Zhou J-J, Zhang J-Z. AUX/IAA gene family in plants: Molecular structure, regulation, and function. Int J Mol Sci. 2018;19: 259. doi: 10.3390/ijms19010259 29337875 PMC5796205

[88] Li S, Yan X, Huang X, Addo-Danso S, Lin S, Zhou L. Physiological differences and transcriptome analysis reveal that high enzyme activity significantly enhances drought tolerance in chinese fir (*Cunninghamia lanceolata*). Forests. 2023;14: 967. doi: 10.3390/f14050967

[89] Souza GB de, Mendes TA de O, Fontes PP, Barros V de A, Gonçalves AB, Ferreira, et al. Genome-wide identification and expression analysis of dormancy-associated gene 1/auxin repressed protein (DRM1/ARP) gene family in *Glycine max*. Prog Biophys Mol Biol. 2019;146: 134–141. doi: 10.1016/j.pbiomolbio.2019.03.006 30914276

[90] Perdomo JA, Capó-Bauçà S, Carmo-Silva E, Galmés J. Rubisco and rubisco activase play an important role in the biochemical limitations of photosynthesis in rice, wheat, and maize under high temperature and water deficit. Front Plant Sci. 2017;8: 490. doi: 10.3389/fpls.2017.00490 28450871 PMC5390490

[91] Drake JE, Power SA, Duursma RA, Medlyn BE, Aspinwall MJ, Choat B, et al. Stomatal and non-stomatal limitations of photosynthesis for four tree species under drought: A comparison of model formulations. Agric For Meteorol. 2017;247: 454–466. doi: 10.1016/j.agrformet.2017.08.026

[92] Lei P, Liu Z, Li J, Jin G, Xu L, Ji X, et al. Integration of the physiology, transcriptome and proteome reveals the molecular mechanism of drought tolerance in *Cupressus gigantea*. Forests. 2022;13: 401. doi: 10.3390/f13030401

[93] Foyer CH, Neukermans J, Queval G, Noctor G, Harbinson J. Photosynthetic control of electron transport and the regulation of gene expression. J Exp Bot. 2012;63: 1637–1661. doi: 10.1093/jxb/ers013 22371324

[94] Johnson JE, Berry JA. The role of Cytochrome b6f in the control of steady-state photosynthesis: a conceptual and quantitative model. Photosynth Res. 2021;148: 101–136. doi: 10.1007/s11120-021-00840-4 33999328 PMC8292351

[95] Shimakawa G, Miyake C. Oxidation of P700 ensures robust photosynthesis. Front Plant Sci. 2018;9. doi: 10.3389/fpls.2018.01617 30459798 PMC6232666

[96] Zlobin IE, Kartashov AV, Pashkovskiy PP, Ivanov YV, Kreslavski VD, Kuznetsov VV. Comparative photosynthetic responses of Norway spruce and Scots pine seedlings to prolonged water deficiency. J Photochem Photobiol B. 2019;201: 111659. doi: 10.1016/j.jphotobiol.2019.111659 31698219

[97] Schiop ST, Al Hassan M, Sestras AF, Boscaiu M, Sestras RE, Vicente O. Biochemical responses to drought, at the seedling stage, of several Romanian Carpathian populations of Norway spruce (*Picea abies* L. Karst). Trees. 2017;31: 1479–1490. doi: 10.1007/s00468-017-1563-1

[98] Granda E, Camarero JJ. Drought reduces growth and stimulates sugar accumulation: new evidence of environmentally driven non-structural carbohydrate use. Tree Physiol. 2017;37: 997–1000. doi: 10.1093/treephys/tpx097 28903526

[99] Piper FI, Fajardo A, Hoch G. Single-provenance mature conifers show higher non-structural carbohydrate storage and reduced growth in a drier location. Tree Physiol. 2017;37: 1001–1010. doi: 10.1093/treephys/tpx061 28549182

[100] Gall HL, Philippe F, Domon J-M, Gillet F, Pelloux J, Rayon C. Cell wall metabolism in response to abiotic stress. Plants. 2015;4: 112–166. doi: 10.3390/plants4010112 27135320 PMC4844334

[101] de Jesús-Pires C, Ferreira-Neto JRC, Pacifico Bezerra-Neto J, Kido EA, de Oliveira Silva RL, Pandolfi V, et al. Plant thaumatin-like proteins: function, evolution and biotechnological applications. Curr Protein Pept Sci. 2020;21: 36–51. doi: 10.2174/1389203720666190318164905 30887921

[102] Laur J, Hacke UG. Exploring *Picea glauca* aquaporins in the context of needle water uptake and xylem refilling. New Phytol. 2014;203: 388–400. doi: 10.1111/nph.12806 24702644

[103] Cheng Z, Zhang X, Yao W, Gao Y, Zhao K, Guo Q, et al. Genome-wide identification and expression analysis of the xyloglucan endotransglucosylase/hydrolase gene family in poplar. BMC Genomics. 2021;22: 804. doi: 10.1186/s12864-021-08134-8 34749656 PMC8576992

[104] Hartmann H, Trumbore S. Understanding the roles of nonstructural carbohydrates in forest trees—from what we can measure to what we want to know. New Phytol. 2016;211: 386–403. doi: 10.1111/nph.13955 27061438

[105] Jeandet P, Formela-Luboińska M, Labudda M, Morkunas I. The role of sugars in plant responses to stress and their regulatory function during development. Int J Mol Sci. 2022;23: 5161. doi: 10.3390/ijms23095161 35563551 PMC9099517

[106] Ivanov YV, Kartashov AV, Zlobin IE, Sarvin B, Stavrianidi AN, Kuznetsov VV. Water deficit-dependent changes in non-structural carbohydrate profiles, growth and mortality of pine and spruce seedlings in hydroculture. Environ Exp Bot. 2019;157: 151–160. doi: 10.1016/j.envexpbot.2018.10.016

[107] Figueroa-Macías JP, García YC, Núñez M, Díaz K, Olea AF, Espinoza L. Plant growth-defense trade-offs: molecular processes leading to physiological changes. Int J Mol Sci. 2021;22: 693. doi: 10.3390/ijms22020693 33445665 PMC7828132

[108] Neuser J, Metzen CC, Dreyer BH, Feulner C, van Dongen JT, Schmidt RR, et al. HBI1 mediates the trade-off between growth and immunity through its impact on apoplastic ROS homeostasis. Cell Rep. 2019;28: 1670–1678.e3. doi: 10.1016/j.celrep.2019.07.029 31412238

[109] Kim HU. Lipid metabolism in plants. Plants. 2020;9: 871. doi: 10.3390/plants9070871 32660049 PMC7411677

[110] Hoch G, Richter A, Körner C. Non-structural compounds in temperate forest trees. Plant Cell Environ. 2003;26: 1067–1081. doi: 10.1046/j.0016-8025.2003.01032.x

[111] Tomasella M, Petrussa E, Petruzzellis F, Nardini A, Casolo V. The possible role of non-structural carbohydrates in the regulation of tree hydraulics. Int J Mol Sci. 2019;21: 144. doi: 10.3390/ijms21010144 31878253 PMC6981889

[112] Kobayashi K, Endo K, Wada H. Roles of lipids in photosynthesis. In: Nakamura Y, Li-Beisson Y, editors. Lipids in plant and algae development. Cham: Springer International Publishing; 2016. pp. 21–49. doi: 10.1007/978-3-319-25979-6_2 27023230

[113] Walker RP, Chen Z-H, Famiani F. Gluconeogenesis in plants: a key interface between organic acid/amino acid/lipid and sugar metabolism. Molecules. 2021;26: 5129. doi: 10.3390/molecules26175129 34500562 PMC8434439

[114] Mukarram M, Choudhary S, Kurjak D, Petek A, Khan MMA. Drought: sensing, signalling, effects and tolerance in higher plants. Physiol Plant. 2021;172: 1291–1300. doi: 10.1111/ppl.13423 33847385

[115] Chan Z, Yokawa K, Kim W-Y, Song C-P. Editorial: ROS regulation during plant abiotic stress responses. Front Plant Sci. 2016;7. doi: 10.3389/fpls.2016.01536 27807438 PMC5069404

[116] Bilska K, Wojciechowska N, Alipour S, Kalemba EM. Ascorbic acid-the little-known antioxidant in woody plants. Antioxid Basel Switz. 2019;8: 645. doi: 10.3390/antiox8120645 31847411 PMC6943661

[117] Corpas FJ, González-Gordo S, Palma JM. Plant peroxisomes: a factory of reactive species. Front Plant Sci. 2020;11. doi: 10.3389/fpls.2020.00853 32719691 PMC7348659

[118] Sofo A, Scopa A, Nuzzaci M, Vitti A. Ascorbate peroxidase and catalase activities and their genetic regulation in plants subjected to drought and salinity stresses. Int J Mol Sci. 2015;16: 13561–13578. doi: 10.3390/ijms160613561 26075872 PMC4490509

[119] Vaish S, Gupta D, Mehrotra R, Mehrotra S, Basantani MK. Glutathione S-transferase: a versatile protein family. 3 Biotech. 2020;10: 321. doi: 10.1007/s13205-020-02312-3 32656054 PMC7320970

[120] Zhang Y, Diao S, Ding X, Sun J, Luan Q, Jiang J. Transcriptional regulation modulates terpenoid biosynthesis of *Pinus elliottii* under drought stress. Ind Crops Prod. 2023;202: 116975. doi: 10.1016/j.indcrop.2023.116975

[121] Pandian BA, Sathishraj R, Djanaguiraman M, Prasad PVV, Jugulam M. Role of cytochrome P450 enzymes in plant stress response. Antioxidants. 2020;9: 454. doi: 10.3390/antiox9050454 32466087 PMC7278705

[122] Tahmasebi A, Niazi A, Akrami S. Integration of meta-analysis, machine learning and systems biology approach for investigating the transcriptomic response to drought stress in *Populus* species. Sci Rep. 2023;13: 847. doi: 10.1038/s41598-023-27746-6 36646724 PMC9842770

[123] Rao MJ, Xu Y, Tang X, Huang Y, Liu J, Deng X, et al. CsCYT75B1, a citrus cytochrome P450 gene, is involved in accumulation of antioxidant flavonoids and induces drought tolerance in transgenic *Arabidopsis*. Antioxid Basel Switz. 2020;9: 161. doi: 10.3390/antiox9020161 32079281 PMC7070963

[124] Kampinga HH, Craig EA. The HSP70 chaperone machinery: J proteins as drivers of functional specificity. Nat Rev Mol Cell Biol. 2010;11: 579–592. doi: 10.1038/nrm2941 20651708 PMC3003299

[125] Gautam R, Meena RK, Rampuria S, Shukla P, Kirti PB. Ectopic expression of DnaJ type-I protein homolog of *Vigna aconitifolia* (VaDJI) confers ABA insensitivity and multiple stress tolerance in transgenic tobacco plants. Front Plant Sci. 2023;14: 1135552. doi: 10.3389/fpls.2023.1135552 37152162 PMC10154610

[126] Liu Y, Song Q, Li D, Yang X, Li D. Multifunctional roles of plant dehydrins in response to environmental stresses. Front Plant Sci. 2017;8: 1018. doi: 10.3389/fpls.2017.01018 28649262 PMC5465263

[127] Perdiguero P, Barbero MC, Cervera MT, Soto A, Collada C. Novel conserved segments are associated with differential expression patterns for *Pinaceae* dehydrins. Planta. 2012;236: 1863–1874. doi: 10.1007/s00425-012-1737-4 22922940

[128] Liu J-J, Ekramoddoullah AKM. The CC-NBS-LRR subfamily in Pinus monticola: targeted identification, gene expression, and genetic linkage with resistance to *Cronartium ribicola*. Phytopathology. 2007;97: 728–736. doi: 10.1094/PHYTO-97-6-0728 18943604

[129] Van Ghelder C, Parent GJ, Rigault P, Prunier J, Giguère I, Caron S, et al. The large repertoire of conifer NLR resistance genes includes drought responsive and highly diversified RNLs. Sci Rep. 2019;9: 11614. doi: 10.1038/s41598-019-47950-7 31406137 PMC6691002

[130] Cobo-Simón I, Maloof JN, Li R, Amini H, Méndez-Cea B, García-García I, et al. Contrasting transcriptomic patterns reveal a genomic basis for drought resilience in the relict fir *Abies pinsapo* Boiss. Tree Physiol. 2023;43: 315–334. doi: 10.1093/treephys/tpac115 36210755

[131] Wang M, Ren L-T, Wei X-Y, Ling Y-M, Gu H-T, Wang S-S, et al. NAC transcription factor TwNAC01 positively regulates drought stress responses in *Arabidopsis* and *Triticale*. Front Plant Sci. 2022;13. doi: 10.3389/fpls.2022.877016 35812952 PMC9257188

[132] Lu D, Wang T, Persson S, Mueller-Roeber B, Schippers JHM. Transcriptional control of ROS homeostasis by KUODA1 regulates cell expansion during leaf development. Nat Commun. 2014;5: 3767. doi: 10.1038/ncomms4767 24806884 PMC4024751

[133] Agarwal P, Mitra M, Banerjee S, Roy S. MYB4 transcription factor, a member of R2R3-subfamily of MYB domain protein, regulates cadmium tolerance via enhanced protection against oxidative damage and increases expression of PCS1 and MT1C in *Arabidopsis*. Plant Sci. 2020;297: 110501. doi: 10.1016/j.plantsci.2020.110501 32563471

[134] Wyrzykowska A, Bielewicz D, Plewka P, Sołtys-Kalina D, Wasilewicz-Flis I, Marczewski W, et al. The MYB33, MYB65, and MYB101 transcription factors affect *Arabidopsis* and potato responses to drought by regulating the ABA signaling pathway. Physiol Plant. 2022;174: e13775. doi: 10.1111/ppl.13775 36050907 PMC9828139

[135] Kitajima S, Koyama T, Ohme-Takagi M, Shinshi H, Sato F. Characterization of gene expression of NsERFs, transcription factors of basic PR genes from *Nicotiana sylvestris*. Plant Cell Physiol. 2000;41: 817–824. doi: 10.1093/pcp/41.6.817 10945353

[136] Zhang J, Wang D, Chen P, Zhang C, Yao S, Hao Q, et al. The transcriptomic analysis of the response of *Pinus massoniana* to drought stress and a functional study on the ERF1 transcription factor. Int J Mol Sci. 2023;24: 11103. doi: 10.3390/ijms241311103 37446285 PMC10342239

[137] Rehman S, Mahmood T. Functional role of DREB and ERF transcription factors: regulating stress-responsive network in plants. Acta Physiol Plant. 2015;37: 178. doi: 10.1007/s11738-015-1929-1

[138] Sun Y, Oh D-H, Duan L, Ramachandran P, Ramirez A, Bartlett A, et al. Divergence in the ABA gene regulatory network underlies differential growth control. Nat Plants. 2022;8: 549–560. doi: 10.1038/s41477-022-01139-5 35501452

[139] Hsu K-H, Liu C-C, Wu S-J, Kuo Y-Y, Lu C-A, Wu C-R, et al. Expression of a gene encoding a rice RING zinc-finger protein, OsRZFP34, enhances stomata opening. Plant Mol Biol. 2014;86: 125–137. doi: 10.1007/s11103-014-0217-6 25002225

[140] Ding S, Zhang B, Qin F. Arabidopsis RZFP34/CHYR1, a ubiquitin E3 ligase, regulates stomatal movement and drought tolerance via SnRK2.6-mediated phosphorylation. Plant Cell. 2015;27: 3228–3244. doi: 10.1105/tpc.15.00321 26508764 PMC4682294

[141] Schueler S, George J-P, Karanitsch-Ackerl S, Mayer K, Klumpp RT, Grabner M. Evolvability of drought response in four native and non-native conifers: opportunities for forest and genetic resource management in Europe. Front Plant Sci. 2021;12: 648312. doi: 10.3389/fpls.2021.648312 34305960 PMC8295755

