# Supplementary figures and images for: De novo transcriptome assembly and discovery of drought-responsive genes in white spruce (Picea glauca)
Source: PLoS One. 2025 Jan 3;20(1):e0316661. doi: 10.1371/journal.pone.0316661 (PMC11698436; doi:10.1371/journal.pone.0316661)

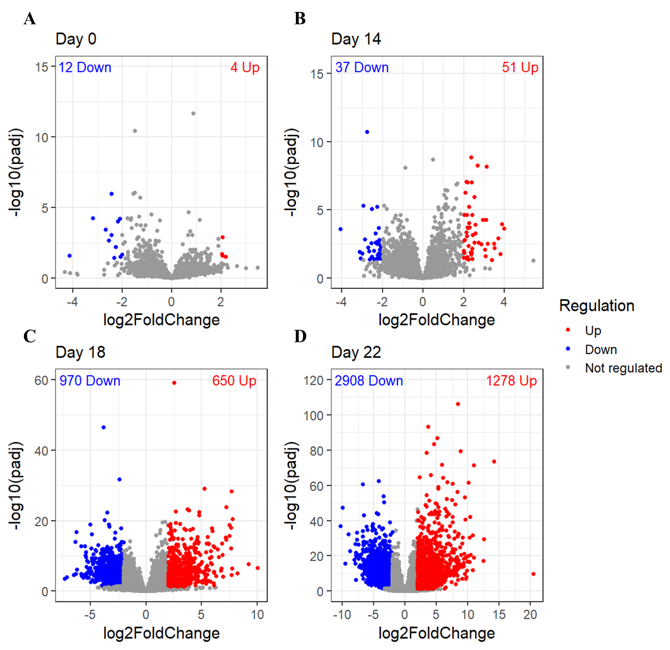

Supplement: S1 Fig — Volcano plots of DEGs are presented for days 0, 14, 18 and 22 (A-D). Unigenes in blue are under-represented in stressed seedlings (FDR ⩽ 0.05 and a log2FC ⩽ -2), while transcripts in red are over-represented in stressed seedlings (FDR ⩽ 0.05 and a log2FC ⩾ 2). Unigenes in gray are not significantly different between the two groups. (TIF) [file pone.0316661.s001.tif]

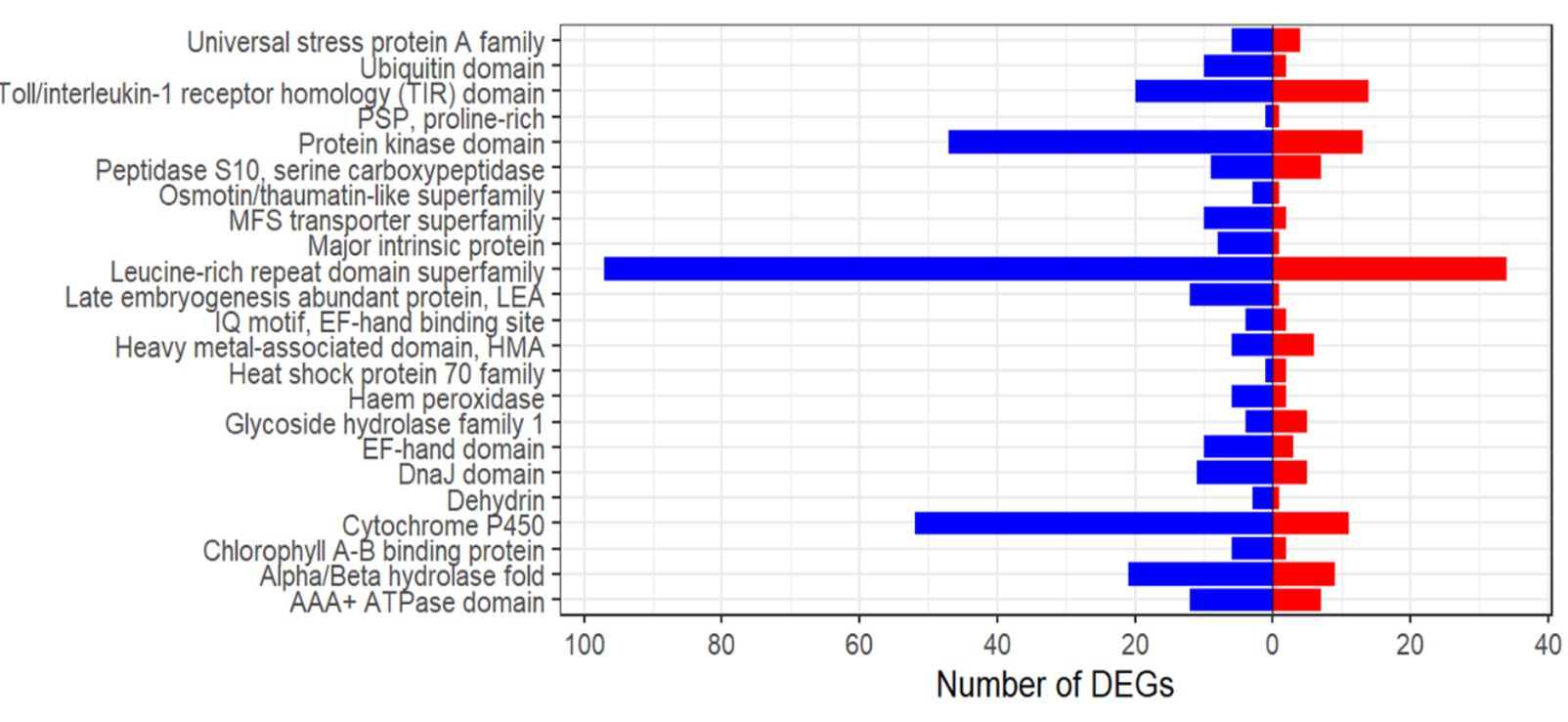

Supplement: S2 Fig — The plot represents the number of up- and down-regulated DEGs at day 22 encoding selected protein families with known roles in plant drought response. (TIF) [file pone.0316661.s002.tif]

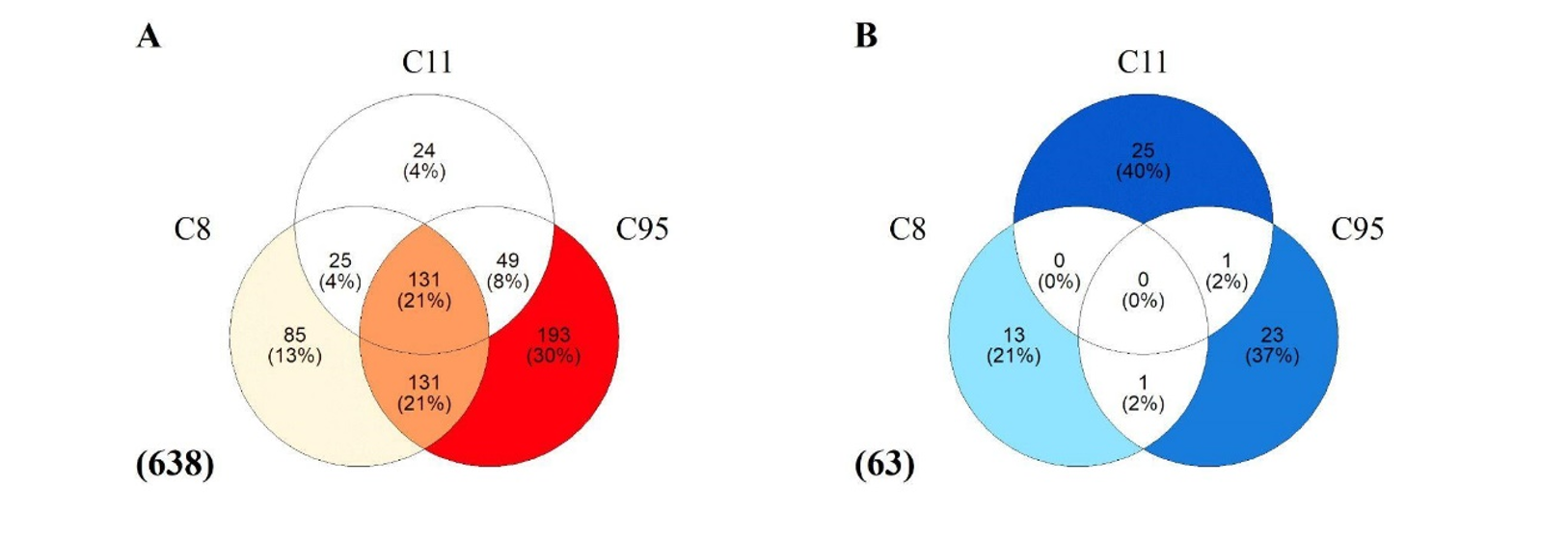

Supplement: S3 Fig — The Venn diagrams depict the overlaps of (A) up-regulated (red) and (B) down-regulated (blue) DEGs across the C8, C11, and C95 clones. The total number of DEGs are shown in bold brackets. (TIF) [file pone.0316661.s003.tif]

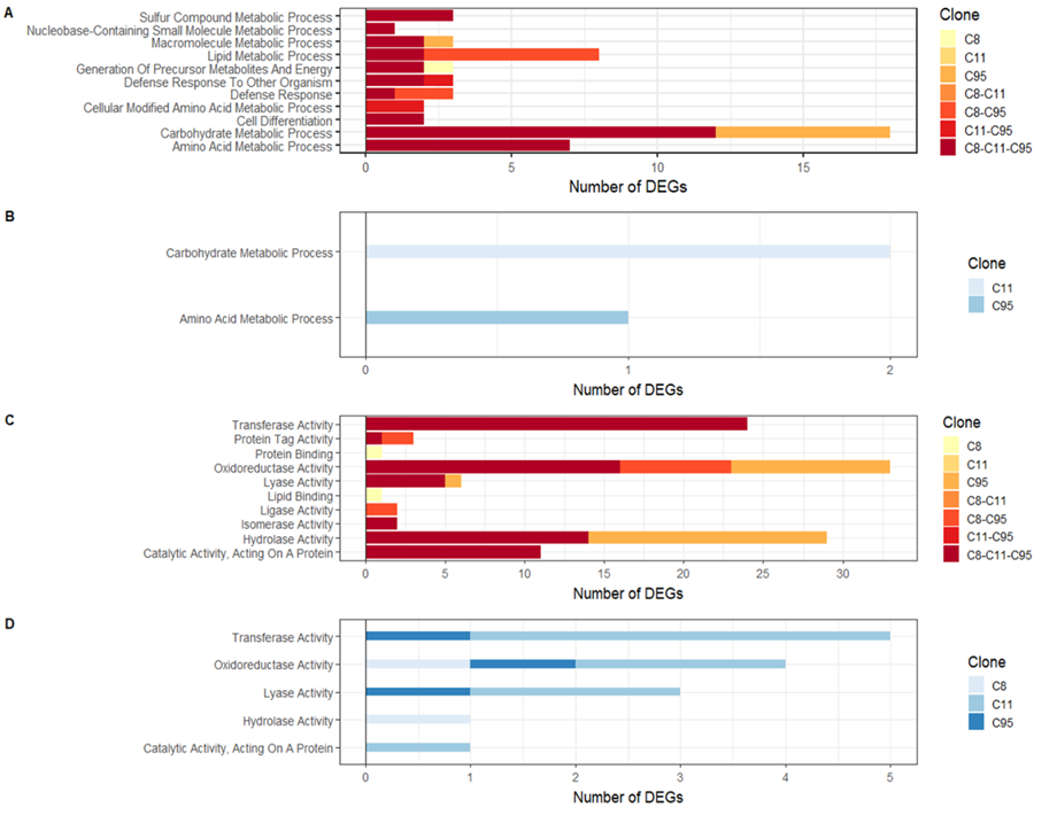

Supplement: S4 Fig — GO annotation of biological process (BP) of (A) up- and (B) down-regulated DEGs of clones and GO annotation of molecular functions (MF) of (C) up- and (D) down-regulated DEGs. Unique DEGs correspond to C8, C11 or C95 and the shared DEGs among clones correspond to C8-C11, C11-C95 and C8-C11-C95. (TIF) [file pone.0316661.s004.tif]
