# Supplementary material for: De novo transcriptome assembly and discovery of drought-responsive genes in white spruce (Picea glauca)
Source: PLoS One. 2025 Jan 3;20(1):e0316661. doi: 10.1371/journal.pone.0316661 (PMC11698436; doi:10.1371/journal.pone.0316661)
Supplement: S1 Methods — (DOCX) [file pone.0316661.s015.docx]

**Supplementary Methods S1**

**Methodological details of experiences used for *de novo* transcriptome assembly**

1. **Experiment 1**

Samples from this experiment were used to perform the *de novo* transcriptome assembly (n=8 stressed and n=8 control).

**1.1. Study site, water treatment and plant material**

Samples were obtained from an experimental garden established in 2013 at the Ontario Forest Research Institute Nursery and Arboretum in Sault Ste. Marie, Ont, Canada (46.546501°N, -84.45565°W, 220 m a.s.l.). Water treatment began in the spring of 2014 and consisted of rainfall exclusion and irrigation treatments. The rainfall exclusion system consisted of a network of gutters that removed ~25% of the surrounding canopy, and the irrigation treatment consisted of 25 mm of water per week using overhead sprinklers from June to August. Trees were arranged in a randomized complete block design. We sampled needles from 3 trees in 8 blocks to have 12 irrigated and 12 rainfall exclusion samples. Sampling was performed at 10 am in early September 2021. Foliage samples were frozen in liquid nitrogen immediately after removal from the trees and stored at -70 °C. Further details of the experimental garden can be found in the publication by Belluau et al. (2021) [1].

**1.2. RNA extraction, RNA-seq libraries synthesis and sequencing**

RNA was extracted by grinding 15 to 20 mg of tissue to a fine powder in liquid nitrogen and using Promega's ReliaPrep™ RNA Tissue Miniprep System, which uses guanidine thiocyanate (GTC) and 1-thioglycerol. Total RNA concentration was determined using a Thermo Scientific™ NanoDrop™ OneC (http://www.nanodrop.com/support) and stored at -70°C. The 16 samples with the best nucleic acid ratio (260/280 and 260/230) were selected for sequencing, providing 8 control samples and 8 stressed samples with good representation of the block design sampling. Quality assessment, RNA-seq libraries and sequencing were performed by the Genome Quebec Innovation Center at McGill University (Montreal, Quebec, Canada). Sequencing was performed on an Illumina NovaSeq6000 S4 sequencing lane (Pair End, 2x100pb).

1. **Experiment 2**

Samples from this experiment were used to perform the *de novo* transcriptome assembly (n=6 stressed).

**2.1. Plant material**

Four-year-old white spruce seedlings were placed in a greenhouse in Chicoutimi, Canada, in the spring of 2015 and exposed to control and defoliation treatments by spruce budworms (*Choristoneura fumiferana*). A total of 47 plants were used as either controls (n = 30) or defoliated treatments (n = 17). For the defoliation treatment, 60 second-stage larval spruce budworms (*Choristoneura fumiferana*) were placed on each sapling (see Deslauriers et al. 2019 for details [2]). Foliage of the year was then sampled six and four times for control and defoliation treatment saplings, respectively, between days 154 and 258 of the year (no foliage of the year remained for the defoliation treatment in the last two samplings). Foliage samples were frozen in liquid nitrogen immediately after removal from the trees and stored at -80°C. Needles were ground to powder using a MixerMill 300 (Retsch. http://www.retsch.com/) and nitrogen-cooled steel grinding balls. Powdered foliage tissue was stored at -80°C until RNA extraction.

**2.2. RNA extraction, cDNA preparation and sequencing**

RNA extraction and cDNA preparation conditions were performed as described by Stival Sena et al. (2018) [3]. 500 ng of RNA per sample was used to synthesize mRNA libraries using the TruSeq® Stranded mRNA Kit (Illumina Canada Inc., Victoria, BC, Canada), following the manufacturer's protocol with a few modifications. First, custom adapters compatible with the Illumina technology were synthesized (IDT, Coralville, IA, USA) and their concentration was halved at 25 nM per reaction to avoid adapter dimer molecules, and Tris-NaCl (10 mM. 50 mM) was used at 25 nM to complete the volume. Second, strand synthesis, label clean-up and library amplification clean-up were performed using the Axygen® AxyPrep™ Mag PCR Clean-Up Kit (Axygen Biosciences, Union City, CA, USA), while post-ligation clean-up was performed using a 0.85 ratio of PEG/NaCl SPRI® solution on beads containing adapter-ligated DNA. Quantification of each library was determined using a Nanodrop ND-1000 (Thermo Scientific, Wilmington, DE, USA), and library fragment size distribution and absence of dimer molecules were verified using an Agilent Bioanalyzer 2100 with High Sensitivity DNA Chips (Agilent Technologies Inc., Santa Clara, CA, USA).

Six pools of 19 libraries at equimolar concentration (114 libraries in total) were prepared and purified according to the library amplification cleanup protocol described in the library preparation protocol. The libraries included in these pools were the 47 white spruce libraries for this publication, and 66 libraries for the rest of the experiment with black spruce or balsam fir (see Deslauriers et al. (2019) for a description of the full experiment). The concentration and fragment size distribution of the pools were evaluated using a Nanodrop ND1000 and an Agilent Bioanalyzer 2100, respectively. The six pools were sequenced at the Genome Quebec Innovation Centre at McGill University (Montreal, Quebec, Canada) on an Illumina HiSeq 2500 sequencing system (2 × 250 bp), one pool per run.

**3. Experiment 3**

This experiment was carried out as described by Stival Sena *et al*. (2018) [3]. Samples from this experiment were used to perform the *de novo* transcriptome assembly (n=3 stressed and n=3 control) and for the transcriptomic analysis presented in this study (n=48).

**3.1. Plant material**

Seedlings of three white spruce clones (C8, C11 and C95), produced by somatic embryogenesis and grown in containers for two years, were subjected to two irrigation treatments: well-watered (control) and drought (no water). Young needles were sampled at five time points (0, 7, 14, 18 and 22 days) from the start of the treatments. Two plants per clone (replicates) in both irrigation treatments were sampled at each sampling time point (60 plants in total). In the present study, only four time points (0, 14, 18 and 22) were used because no differentially expressed genes were observed at day 7 (n=48). Foliage samples were frozen in liquid nitrogen immediately after removal from the trees and stored at -80°C. Needles were ground to powder using a MixerMill 300 (Retsch. http://www.retsch.com/) and nitrogen-cooled steel grinding balls. Powdered foliage tissue was stored at -80°C until RNA extraction. Further details of the experiment can be found in the methods of Stival Sena *et al*. (2018) [3].

**3.2. RNA extraction and cDNA preparation**

RNA was extracted by grinding tissues in liquid nitrogen to a fine powder and by utilizing the cetyltrimethyl ammonium bromide (CTAB) extraction method as described by Chang et al. (1993) [4], with modifications [5]. The total RNA concentration was determined using a NanoDrop 1000 (Thermo Scientific. http://www.thermoscientific.com/) and assessed for quality with an Agilent 2100 Bioanalyzer and Agilent RNA 6000 Nano Kit LabChips (Agilent Technologies Inc., http://www.agilent.com/) and stored at −80 °C. Complementary DNAs were prepared from 500 ng of total RNA using the Quantitect Reverse Transcription Kit (Qiagen, Germantown, MD, USA) and then diluted 1:4 in RNase-free water.

**3.3. Libraries and sequencing**

We used 500 ng of RNA per sample to synthesize mRNA libraries using the TruSeq® Stranded mRNA Kit (Illumina Canada Inc., Victoria, BC, Canada), following the manufacturer's protocol with a few modifications. First, custom adapters compatible with the Illumina technology were synthesized (IDT, Coralville, IA, USA) and their concentration was halved at 25 nM per reaction to avoid adapter dimer molecules, and Tris-NaCl (10 mM. 50 mM) was used at 25 nM to complete the volume. Second, strand synthesis, label clean-up and library amplification clean-up were performed using the Axygen® AxyPrep™ Mag PCR Clean-Up Kit (Axygen Biosciences, Union City, CA, USA), while post-ligation clean-up was performed using a 0.85 ratio of PEG/NaCl SPRI® solution on beads containing adapter-ligated DNA. Quantification of each library was determined using a Nanodrop ND-1000 (Thermo Scientific, Wilmington, DE, USA), and library fragment size distribution and absence of dimer molecules were verified using an Agilent Bioanalyzer 2100 with High Sensitivity DNA Chips (Agilent Technologies Inc., Santa Clara, CA, USA). An equimolar pool of 59 libraries was prepared and purified according to the library amplification cleanup protocol as described in the library construction protocol. The concentration and fragment size distribution of the pool were evaluated using a Nanodrop ND1000 and an Agilent Bioanalyzer 2100, respectively. The pool was sequenced using the rapid run method (2 × 250 bp) at the Genome Quebec Innovation Centre at McGill University (Montreal, Quebec, Canada) using an Illumina HiSeq 2500 sequencing system.

**3.4. Data filtering**

Raw sequencing produced a total of 120 FASTQ files representing 148 Gbases in 592 million 2x125 bp reads. Filtering retained 114.6 Gbases (74.7% bases) in 551.6 million HQ sequences (93.1% sequences), of which 495.6 million (89.9%) are Paired-End (both reads from paired sequencing yielded a HQ segment. Read counts were obtained for 37,491 genes and 36,701 of these genes had at least one read count. For more information, see the publication of Stival Sena *et al*. (2018) [3].

**References**

1. Belluau M, Vitali V, Parker WC, Paquette A, Messier C. Overyielding in young tree communities does not support the stress-gradient hypothesis and is favoured by functional diversity and higher water availability. J Ecol. 2021;109: 1790–1803. doi:10.1111/1365-2745.13602

2. Deslauriers A, Fournier M-P, Cartenì F, Mackay J. Phenological shifts in conifer species stressed by spruce budworm defoliation. Tree Physiol. 2019;39: 590–605. doi:10.1093/treephys/tpy135

3. Stival Sena J, Giguère I, Rigault P, Bousquet J, Mackay J. Expansion of the dehydrin gene family in the *Pinaceae* is associated with considerable structural diversity and drought-responsive expression. Tree Physiol. 2018;38: 442–456. doi:10.1093/treephys/tpx125

4. Chang S, Puryear J, Cairney J. A simple and efficient method for isolating RNA from pine trees. Plant Mol Biol Report. 1993;11: 113–116. doi:10.1007/BF02670468

5. Pavy N, Pelgas B, Beauseigle S, Blais S, Gagnon F, Gosselin I, et al. Enhancing genetic mapping of complex genomes through the design of highly-multiplexed SNP arrays: application to the large and unsequenced genomes of white spruce and black spruce. BMC Genomics. 2008;9: 21. doi:10.1186/1471-2164-9-21
